# Electrochemical tracing of hypoxia glycolysis by carbon nanotube sensors, a new hallmark for intraoperative detection of suspicious margins to breast neoplasia

**DOI:** 10.1002/btm2.10236

**Published:** 2021-06-14

**Authors:** Zohreh Sadat Miripour, Fereshteh Abbasvandi, Parisa Aghaee, Sahar NajafiKhoshnoo, Mahsa Faramarzpour, Pooneh Mohaghegh, Parisa Hoseinpour, Naser Namdar, Morteza Hassanpour Amiri, Hadi Ghafari, Sarah Zareie, Fatemeh Shojaeian, Hassan Sanati, Mahna Mapar, Nastaran Sadeghian, Mohammad Esmaeil Akbari, Mohammad Ali Khayamian, Mohammad Abdolahad

**Affiliations:** ^1^ Nano Bio Electronic Devices Lab, School of Electrical and Computer Engineering, College of Engineering University of Tehran Tehran Iran; ^2^ Nano Electronic Center of Excellence, Thin Film and Nanoelectronics Lab, School of Electrical and Computer Engineering, College of Engineering University of Tehran Tehran Iran; ^3^ ATMP Department Breast Cancer Research Center, Motamed Cancer Institute, ACECR Tehran Iran; ^4^ SEPAS Pathology Laboratory Tehran Iran; ^5^ Cancer Research Center Shahid Beheshti University of Medical Sciences Tehran Iran; ^6^ Integrative Oncology Department Breast Cancer Research Center, Motamed Cancer Institute, ACECR Tehran Iran; ^7^ Cancer Institute, Imam Khomeini Hospital Tehran University of Medical Sciences Tehran Iran; ^8^ UT&TUMS Cancer Electrotechnique Research Center YAS Hospital, Tehran University of Medical Sciences Tehran Iran

**Keywords:** cancer, clinical trial, diagnostic probe, frozen pathology, hypoxia glycolysis, surgery

## Abstract

For most people, the first step in treatment is to take out the tumor (surgery), so precise and fast diagnosis of any sign of high‐risk and neoplastic cells, especially in surgical cavity margins, is significant. The frozen pathology method is the conventional standard of intraoperative diagnosis, but the low number of slides prepared from non‐fixed tissues prevents us from achieving a perfect diagnosis. Although many improvements in intraoperative margin detection were achieved, still real‐time detection of neoplastic lesions is crucial to improving diagnostic quality. Functionalized carbon nanotubes grown on the electrode needles lively and selectively determine the H_2_O_2_ released from cancer/atypical cells through reverse Warburg effect and hypoxia assisted glycolysis pathways in a quantitative electrochemical manner. The study was carried out on cell lines, 57 in vivo mice models with breast cancer, and 258 fresh in vitro samples of breast cancer tumors. A real‐time electrotechnical system, named cancer diagnostic probe (CDP) (US Patent Pub. No.: US 2018/02991 A1, US 2021/0007638 A1, and US 2021/0022650 A1 [publications], and US 10,786,188 B1 [granted]), has been developed to find pre‐neoplastic/neoplastic cells in vivo in a quantitative electrochemical manner by tracing hypoxia glycolysis byproducts. Matched pathological evaluations with response peaks of CDP were found based on the presence of neoplasia (from atypia to invasive carcinoma) in live breast tissues. The ability of CDP to find neoplastic lesions in mice models in vivo and fresh breast tumors in vitro was verified with sensitivity and specificity of 95% and 97%, respectively. The system may help a surgeon assistant system for usage in the operating room after passing many trials and standard examinations in the future.

## 1 INTRODUCTION

Due to the impact of intraoperative margin detection, scientists attempted to develop new methods, which resulted in many interesting technologies such as MasSpec Pen^1^ (based on mass spectroscopy from the lipids/proteins of margin surface), Margin Probe^2^ (used radiofrequency for stimulating and sensing the margins), and confocal laser endomicroscopy for margin detection of brain tumors.^3^ Some of the reported advantages and limitations of these techniques are demonstrated in Tables S1 and S2. Although many improvements were achieved, still no intraoperative technique has been reported for the detection of surgical margins with pathological classification in breast cancer (as one of the most important onco‐surgeries required to accurate margin detection).

In this paper, a new system based on real‐time tracing the hypoxia glycolysis function of cancer cells was introduced as a potential tool for detecting cancer lesions and especially margin detection.

The mechanism of such pathologically classified diagnosis is based on recording the current peaks of H_2_O_2_ released during three important pathways activated in epithelial cells during cancerous transformation. First, oncogenic stimulation of normal cells followed by DNA damage and oncogene activation as the signs of tumor initiation.^4^ Second, the reverse Warburg effect in which released H_2_O_2_ from pre‐invasive/neoplastic cells in the microenvironment changes the function of tumor‐associated fibroblast (TAF) from aerobic to glycolysis metabolisms.^5^, ^6^ Third, hypoxia assisted glycolysis of tumor cells, as their distinct metabolism respect to normal cells.^7^, ^8^ It has been shown that H_2_O_2_ produced and released by oncogenic stimulated normal cells would result in their transformation to atypical/pre‐invasive phenotypes.^9^ Also, approved reports indicated the strong correlation between activation of hypoxia‐assisted glycolysis and neoplastic transformation of breast cells.^7^, ^8^

This system, named cancer diagnostic probe (CDP), was investigated on wide ranges of human cell lines followed by freshly dissected breast tumors in vitro and then on mice models with breast cancers in vivo. It is the first time that such an electrical diagnoser would be applied as a real‐time cancer detector in live tissues. Here, after recording and calibrating hypoxia based margin diagnosis on 258 fresh breast tumor samples, quantified diagnostic scorings of CDP response peaks were defined in correlation with their permanent histopathology results based on the World Health Organization (WHO) classification of breast tumors.^10^, ^11^, ^12^, ^13^ This diagnostic approach showed more than 95% sensitivity in its best calibration, which shed new light for application as a surgeon assistant in the future after passing clinical trials.

## 2 RESULTS

**FIGURE 1:**
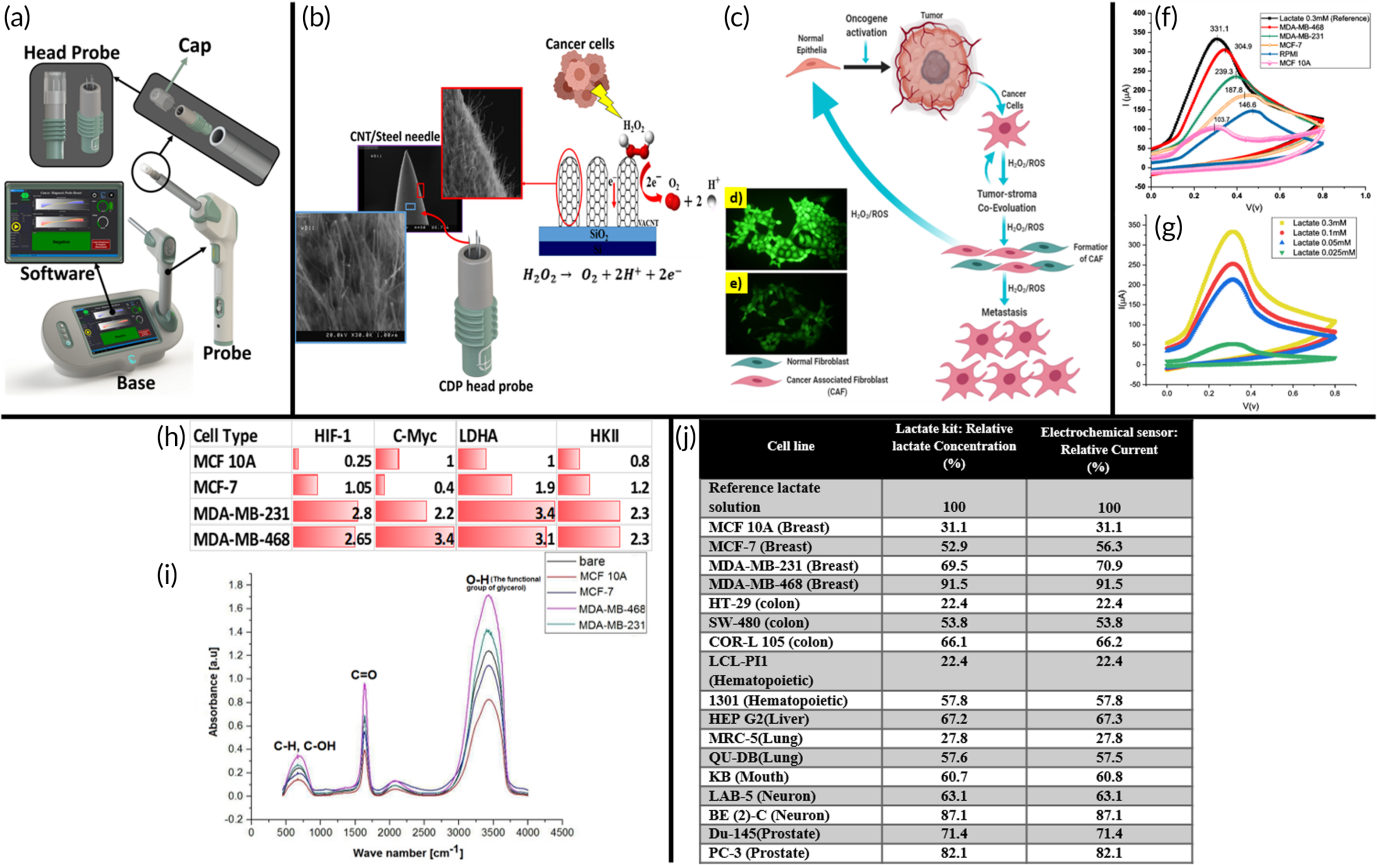
(a) Image of cancer diagnostic probe (CDP) system with a changeable head probe consists of three needle electrodes coated by multiwalled carbon nanotubes (MWCNTs). (b) Selective electrochemical reactions of released H_2_O_2_ on MWCNTs and production of the cathodic ionic peak. The distribution and abundance of nanotubes make a conformal surface for signal extraction, and it is presented in FESEM images. Cancer cells release H_2_O_2_ due to hypoxia assisted glycolysis, as their distinct metabolism respect to normal cells. (c) The reverse Warburg effect also releases H_2_O_2_ from pre‐invasive/neoplastic cells in the microenvironment and changes the function of tumor‐associated fibroblasts from aerobic to glycolysis metabolisms. (d,e) Intracellular reactive oxygen species (e.g., H_2_O_2_) levels were measured using CM‐H2DCFDA fluorescent that emits green fluorescence in the presence of H_2_O_2_, but it decreased in MCF‐7 treated cells (1 × 10^6^) with 6.5 mM of *N*‐acetyl‐l‐cysteine (NAC) as a ROS inhibitor (ab143032). (f) Hypoxia assisted glycolysis related electrochemical cyclic voltammetry cathodic peaks of different phenotypes of breast cell lines (MDA‐MB‐468, MDA‐MB‐231, MCF‐7, and MCF‐10A) in comparison with standard culture media (RPMI) and lactate solution (concentrations of 0.3 mM). The intensity of the peaks is in correlation with the cancerous progression of the cells. (g) CV responses of different concentrations of standard unstable H_2_O_2_ contained solution (with the base of lactate) as a reference sample. (h) RT‐PCR of hypoxia associated transcriptomes from breast cell lines. (i) FTIR was taken from bare RPMI, and the media solution of normal and cancer cell lines. (j) Comparison between the lactate kit and CNT electrochemical assays in normal and breast, colon, neuron, prostate, mouth, lung, liver, and hematopoietic cancer cell lines

CDP consists of an integrated automatic electrochemical readout board and a sensing disposable head probe as the main diagnostic part of the system (US Patent Pub. No.: US 2018/02991 A1, US 2021/0007638 A1, and US 2021/0022650 A1 [publications], and US 10,786,188 B1 [granted]). The sensing head probe was fabricated by the growth of multiwalled carbon nanotubes (MWCNTs) on the tip of steel needles in the conformation of three electrodes, named working (WE), counter (CE), and reference (RE), with a triangular distance of 3 mm from each other (Figure 1a). The head probe was sterilized under plasma sterilizer protocol (standard No: ISO/NP 22441), which did not induce any perturbations on the nanostructures' morphology and function (Figure S1a). The head probe was injected three times into the bare biological sponge to release any weakly bonded CNTs before sterilizing (Figure S1b–d).

Four concerns were considered in choosing the sensing material for CDP. These concerns include biocompatibility, selectivity in sensing ROS/H_2_O_2_, great electrical conductivity, and cheap cost. Carboxylated MWCNTs (C‐MWCNTs) have all of the required parameters for such sensing. Also, previous reports indicated the selective electrochemical sensing abilities of C‐MWCNTs in the detection of H_2_O_2_.^14^, ^15^ CNT has achieved these results covered sensing needles that applied selective interaction with released H_2_O_2_ from abnormal cancer cells (Figure S2a–c) with no post‐recording perturbation on morphology and distributions of the sterilized CNT needles (Figure S2d). The fabricated sensing needles' repeatability was verified by testing more than 200 head probes on similar artificial tissues (Figure S3).

We did many standard biocompatibility tests both in vitro and in vivo (ISO 10993‐5, Part 5: test for in vitro cytotoxicity, ISO 10993‐10, test for irritation and skin sensitization, ISO 10993‐10, test for in vitro inflammation) in both cell lines and animal models with the assistance of Pasteur institute of Iran and received the certificates for in vivo use of the sensor. C‐MWCNTs showed non‐toxicity and non‐inflammatory effects MCF‐10A and HUVEC cell lines (MTT, ANXV/PI) (Figure S4) as well as in New Zealand white rabbit and guinea pig models (tested and approved by Iran Pasteur Institute, Ethics committee approval ID: IR.TUMS.VCR.REC.1397.355) and finally human model (IMED national ID: 14006918495).

It is well known that the release of H_2_O_2_ molecules is one of the sequential evidence during tumor initiation, reverse Warburg effect, and hypoxia assisted glycolysis of cancer cells (Figure 1b,c).^5^, ^6^, ^7^ CDP's detection mechanism has been based on the real‐time breaking of released H_2_O_2_ molecules, and selective releasing of two electrons on CNT covered sensing needles, which resulted in peak current recorded by the readout system (Figure 1b). As a result, a correlation between the cells' pathologic states, the concentration of the generated H_2_O_2_ in the tissue microenvironment, and the electrochemical peak current of the CDP would be observed can be analyzed and calibrated as a diagnostic profile.^16^

**FIGURE 2:**
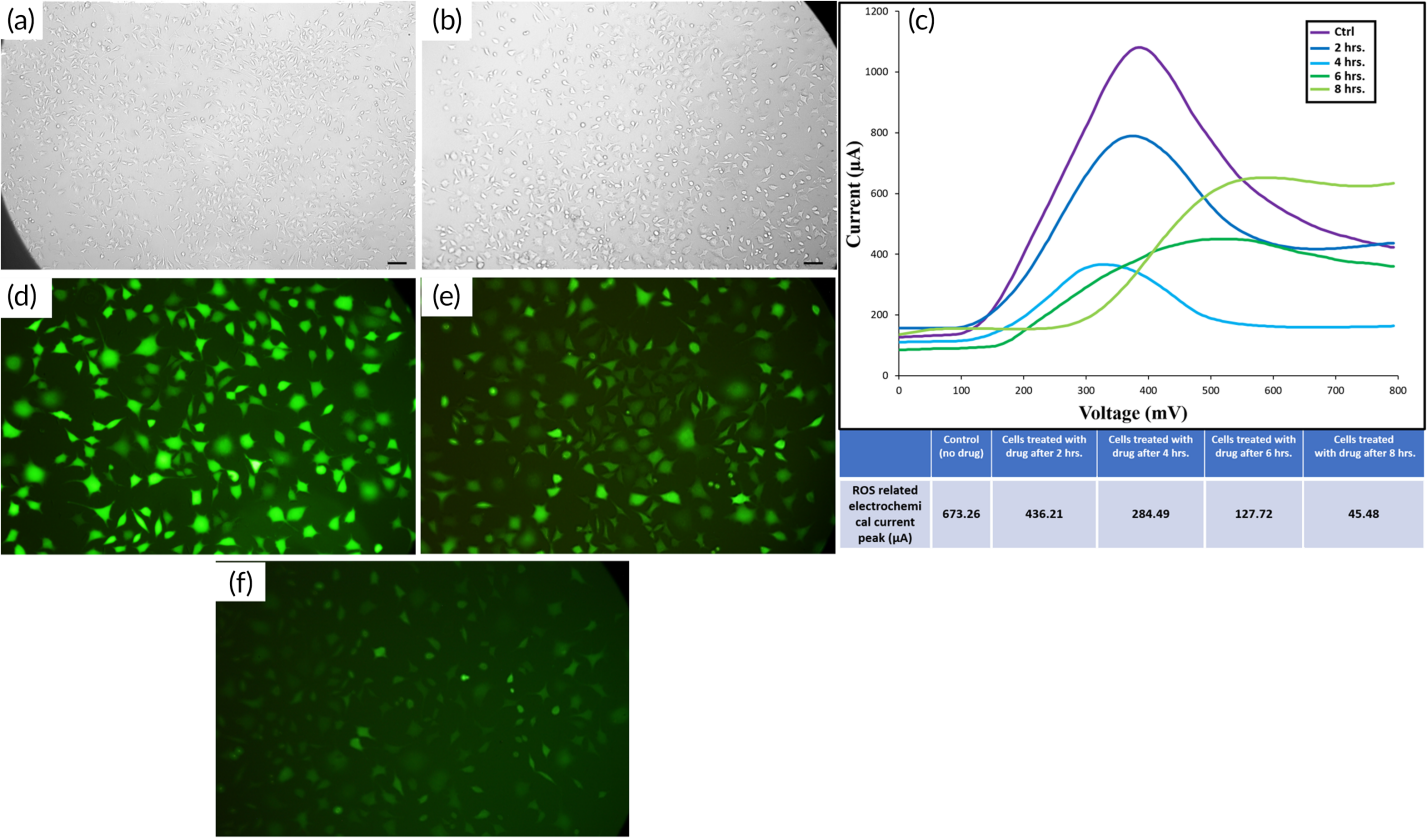
(a) Optical microscope images of the MDA‐MB‐231 breast cancer cell lines before, and (b) after 8 h of the addition of anti‐ROS agent (NAC), (c) electrochemical current diagrams of control (without drug), and the cells treated with NAC drug after 2, 4, 6, and 8 h, Fluorescent microscope image of (d) positive control (100 μM of H_2_O_2_), (e) negative control (without drug), and (f) after 8 h of adding NAC drug

CDP tests on various cell lines (Figure 1f,g and Supplementary Section 1) followed by RT‐PCR, FTIR analysis, and lactate‐based assays confirmed the specific hypoxia‐related responses of CDP, and the investigation of ROS analysis investigated by *N*‐acetyl cysteine (NAC) (see method) (Figure 2).

### 2.1 RT‐PCR analysis

Many reports indicated that pyruvate dehydrogenase inactivation and lactate dehydrogenase activation occurred in hypoxia assisted glycolysis of cancer cells are high in correlation to activation of HIF‐1α, and C‐Myc activation.^17^, ^18^ It is believed that HKII protein mRNA, bound to mitochondria, enhances glucose metabolism through glycolysis in tumors.^19^ HIF‐1α and C‐Myc oncogenes play a crucial role in the upregulation of HKII.^20^, ^21^, ^22^ Another mRNA expressed under hypoxia of tumor cells is associated with the PGAM gene.^23^, ^24^ Activation of this protein would regulate glycolytic flux and adapt cell hypoxia.^25^ Moreover, pyruvate dehydrogenase kinase‐1 is another important mRNA activated by HIF‐1α during hypoxia‐assisted glycolysis. This enzyme induces the inactivation of PDH (the inhibitor of oxidative disposal of pyruvate). Hence, the diversion of the glycolytic flux to lactate production would be increased.^26^ RNA sequencing results of the HIF‐1, C‐Myc, HKII, and LDHA presented in Figure 1h, which indicated the occurrence of hypoxia‐assisted glycolysis in MCF‐7, MDA‐MB‐231, and MDA‐MB‐468 cell lines. Tumor cells expressed a meaningful level of associated hypoxic markers depends on their phenotypic changes to normal cells (MCF‐10A), all calibrated by GAPDH (as housekeeping).

### 2.2 FTIR analysis of breast cell lines' secretion

FTIR spectroscopy was applied to investigate the lactate‐based bonds' presence (including C─H) and oxidative molecules in the media solution of all breast cell lines' phenotypes. Results indicated the increased intensity of C─H, O─H, and molecular bonds due to the invasive progression of cancer cell lines (Figure 1i). The functional group of glycerol, including O─H stretching at 3312 cm^−1^,^27^ was significantly increased in cancer cells' media solution with malignant grades. Also, the C═O bonds located at 1700 cm^−1^ could be attributed to the pyruvate produced by lactate de‐hydrolysis,^28^ which increased in the media of invasive cancer cells as MDA‐MB‐231 and MDA‐MB‐468. C─H and C─OH peaks at 550 cm^−1^ could be attributed to the increased L/P ratio in the breast cancer cells' media solution during progression.

Before determining the in vivo efficacy of CDP in scoring both EMs and IMs (external and internal margins) of mice models and human samples, the selectivity of CDP disposable sensor to ROS/H_2_O_2_ released from cancer cells was investigated by florescent assays before and after the addition of ROS scavenger agent (NAC). In this manner, MDA‐MB‐231 (breast cancer cell line) with a concentration of 10^6^ in DMEM culture solution was used for the test (Figure 2a). NAC (ROS/H_2_O_2_ inhibitor [ab143032] [6.5 mM]) was added to the cells, and all CDP current peaks were recorded before and after the addition of anti‐ROS agent at 2‐h intervals up to 8 h (Figure 2b). Also, ROS‐based fluorescent assay (CM‐H2DCFDA, which emits green fluorescent light in the presence of ROS/H_2_O_2_) as well as optical microscopy imaging was used as standard assays to evaluate the intensity of ROS production and cell viability in correlation with CDP peaks, respectively. After 8 h, the CDP current peaks of the treated cells with NAC were decreased by approximately 15‐fold compared to control cells (without drug), which were strongly correlated with the drastic reduction of ROS‐based green florescent expression (Figure 2). These results were achieved while the cells' viable shapes remained alive (Figure 2a,b). While we know that many other ions and proteins (such as Na, Ca, K, FBS, etc.) exist in the culture solutions, the current peak of CDP just showed a strong reduction after ROS scavenger addition. The intracellular ROS/H_2_O_2_ reduction is comparable to the decrease in the extracellular efflux of ROS/H_2_O_2_ (Figure 2e,f). This test clearly shows the specific effect of the ROS/H_2_O_2_ expression on the CDP current peak, while the optical microscopy images show that the cells have retained their normal morphology and just their ROS generation and secretion activities were decreased.

### 2.3 Applying CDP in tumor diagnosis of mice models

**FIGURE 3:**
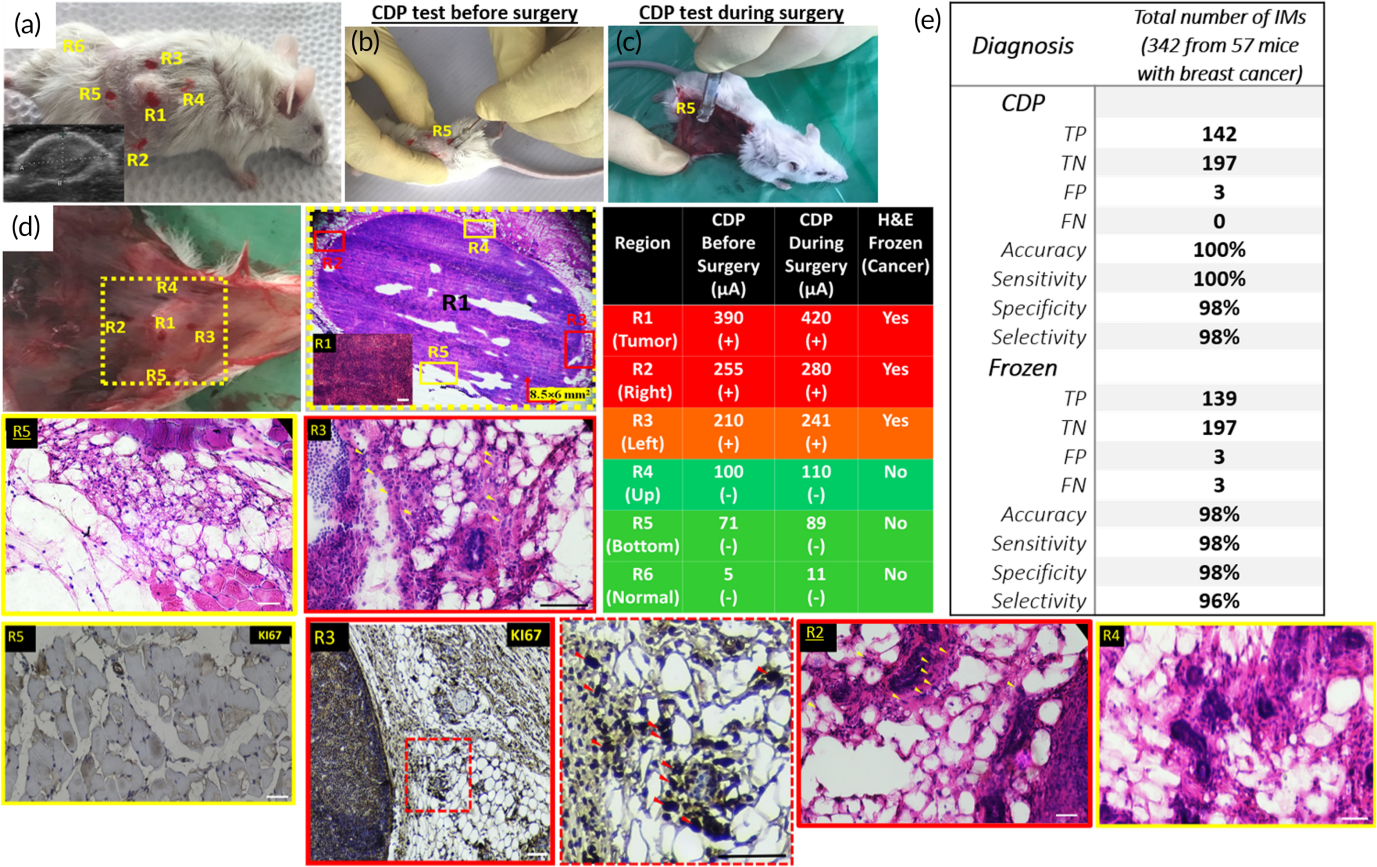
(a) Tumorized mouse by 4T1 breast cancer cell lines checked by sonography (a1). The CDP was tested on tumor and suspicious regions before (b) and during (c) the surgery. (d) The tumor contained suspicious regions were removed and sent for frozen pathology, and the H&E images taken from the center of the tumor (R1) as well as its right (R2), left (R3), up (R4), and bottom (R5) margins, with the distance of 1 mm from the histologically distinct region, were demanded by the CDP results as presented in the table. Green and red marks indicated the normal and cancerous regions with different intensities of filtrated malignant cells through color's sharpness. Before and during surgery, the results of CDP exhibited a significant correlation (99% CI, *p*‐value < 0.00001). Ki67 based IHC assay confirmed the normal state of R5 and cancer involvement in R3 as classifier reference. Triangular arrows in pathological images refer to the cancer cells with hyperchromic and deformed nuclei distributed among the stroma in suspicious regions. (e) Comparative diagnostic parameters of CDP and Frozen pathology vs. permanent pathology as a gold standard

The animal model assay is the first step to determine CDP's in vivo efficiency in scoring both EMs and IMs. All mouse studies were performed according to the ministry of health and medical education protocols for live animals' use and care (Iran National Committee for Ethics in Biomedical Research [IR.TUMS.VCR.REC.1397.355]). In this regard, 60 female mice were tumorized and tested by CDP. Before applying CDP on mice models, their tumors had been distinguished from normal regions by sonography. CDP showed high current intensity (>300 μA vs. <70 μA, *p*‐value < 0.0001) just in tumor locations (Supplementary Section 2, Figure S6). In the next step, individual tumorized mice were candidates for surgery, and CDP checked the boundary lesions beneath their tumors. Pre‐surgical sonography declared the approximate dimension and location of the tumor (e.g., 8.5 × 6 mm^2^: Figure 3a). CDP checked the center of the tumor (R1), four internal boundary regions beneath the tumor (R2‐R5), and a region far from the tumor (R6) before the surgery (Figure 3b). Subsequently, the surgery was started (Figure 3c), and the tumor center and the mentioned regions adjacent to the normal stroma were checked by CDP results showed at least 105 μA differences in current peaks between cancer involved and free lesions (tabled panel in Figure 3) confirmed by frozen and permanent hematoxylin and eosin (H&E). CDP positively scored R1, R2, and R3 as involved regions by neoplastic cells meanwhile it negatively scored R4, R5, and R6 as free regions.

It could be revealed from H&E images (Figure 3d) that R1 was completely a cancer tissue, whereas many cancer cells distributed between the stroma is observable in R2 (yellow triangles in H&E image of R2). R3 consisted of ~40% tumor tissue among normal stroma. Tumor cells have hyperchromic nuclei with increased nucleolus/cytoplasm (N/C) ratio (triangular arrows in H&E images of R2 and R3 regions; Figure 3). R4 and R5 lesions were diagnosed as the normal stroma.

R3 and R5 regions were evaluated by Ki67 immunohistochemistry (IHC) assay as an independent diagnostic marker in correlation with tumor cell proliferation.^29^ Ki67 based IHC classifier identified no trace of cancer proliferation in R5 while confirmed the proliferation of cancer cells in the R3 sample (Figure 3, IHC panels). Table S3 shows the in vivo scoring results of CDP on 57 tumorized mice verified by H&E/IHC assays to indicate its repeatability.

As it is shown in receiver operating characteristic (ROC) analysis and area under the curve (AUC) table, for CDP the area under the curve is 0.992 (*p*‐value < 0.0001 and CI 99% 0.98–1.00) (Figure S7 and Table S6), and this value for frozen pathology was 0.982 (*p*‐value < 0.0001 and CI 99% 0.96–1.00) (Figure S8 and Table S7). The result of the ROC test shows that the CDP has better results than frozen due to the higher area under the curve of CDP (0.992 > 0.982), and it can be used as a diagnostic test of cancerous specimens. The specificity, sensitivity, specificity, and selectivity of CDP and frozen versus permanent pathology as the gold standard are shown in Figure 3e.

### 2.4 Pathological classification of CDP responses on in vitro breast samples

This research aimed to introduce CDP as a real‐time diagnostic tool to find involved body side margins (named IMs) during human cancer surgery. Hence, pathologically confirmed the efficiency of the CDP must be revealed both in vitro and in vivo. All methods for the human subjects were also carried out under relevant guidelines and regulations. They were performed according to the ministry of health and medical education protocols to use human samples. Moreover, patients provided consent according to an ethically approved protocol (IR.TUMS.VCR.REC.1397.355) at our breast cancer central clinics and assistant hospitals.

CDP was applied to record the current peaks of 258 in vitro human fresh samples (biopsied/surgically removed) prepared from 74 breast cancer patients. Tissues were tested immediately after dissection from the body (with a non‐dehydrated weight of 15–25 mg and size of up to 1 cm^2^). All three integrated needle electrodes, assembled on the head probe, were simultaneously entered into the target tissue. The whole process includes replacing and connecting a new head probe (~20 s), entering CDP to the tissue, and recording the data (~15 s due to synchronized real‐time processing) would take place in less than 40 s. The samples' permanent pathological diagnostic results (re‐checked by three histological slides from each sample) were the gold standard in the CDP responses' probable scalability.

**FIGURE 4:**
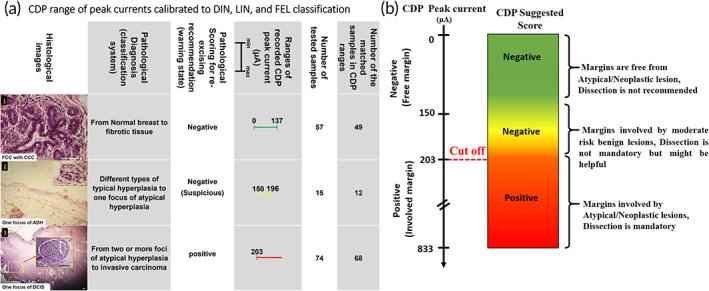
(a) CDP current peak results of 258 in vitro samples from 74 patients associated with pathological diagnosis based on pathological classification (DIN, LIN, and FEL). Normal breast (e.g., breast fatty tissue), UDH (e.g., FCC lesions), and DIN1a (e.g., FCC with CCC and a small focus of ADH) showed response peak ranges from 0 to 196 μA, which CDP negatively scored. DIN1b (e.g., two or more foci of ADH), DIN1c (e.g., low grade‐DCIS), DIN2 (e.g., intermediate DCIS), DIN3 (e.g., high‐grade DCIS), and IDC lesions showed response peaks in the ranges from 203 μA to more than 600 μA which CDP positively scored. (b) Results show meaningful consistency between pathological diagnosis and CDP scoring

Meaningful results were observed after comparing the experimental categorization of samples through their CDP recorded current peaks with their categorization through their H&E pathological diagnoses presented in Figure 4.

Among 258 samples, 26 samples were normal breast stroma, fatty breast tissues, and simple fibroadenoma, which all showed current peaks between 0 and 83 μA. Eighteen samples were non‐proliferative fibrocystic changes (FCC) which all of them showed current peaks between 53 and 111 μA. Ten samples were mild usual ductal hyperplasia (UDH), showed current peaks between 83 and 110 μA. Eleven samples were sclerosis adenosis (SA) in which 8 of them showed current peaks between 86 and 115 μA. Twelve samples were moderate UDH, in which 10 of them showed current peaks between 120 and 137 μA. Nineteen samples were FCC with columnar cell changes (some of them also had one focus suspicious to atypical ductal hyperplasia [ADH]) in which 17 of them showed current peaks between 110 and 173 μA. Thirty‐one of the samples showed involvement in two or more foci of ADH, in which 28 of them showed peak currents between 203 and 250 μA. Twenty‐four of the samples showed involvement in low‐grade ductal carcinoma in situ (DCIS), from 10% to 45% of the histological pattern, in which 21 of those samples showed current peaks between 250 and 360 μA. Twelve samples were invasive ductal carcinoma (IDCs) with distributions between 5% and 55% of histological pattern, which all of them showed current peaks between 610 and 800 μA. Two samples were phyllodes tumor, which both showed peak currents of more than 260 μA. Just 10 samples were found with lobular based atypia/neoplasia. Four of those samples were invasive lobular carcinoma with extensive distribution, which all showed current peaks between 380 and 465 μA. Four samples were atypical lobular hyperplasia, which showed current peaks between 257 and 270 μA. Two other samples were lobular carcinoma in situ, which showed current peaks of about 290 μA. By considering the pathology reports and CDP current peaks of the samples together, a classification of CDP responses was proposed based on the pathological diagnosis. The lowest cut‐off for pathologists on diagnosing a margin as positive (dissection is mandatory) is the presence of at least two foci of ADH.^30^, ^31^ Therefore, 203 μA was proposed as a cut‐off current for the positive scoring of CDP. Samples consisted of ADH with more than two foci, DCIS and IDCs most often showed peak currents higher than 203 μA, while others consisted of one focus of ADH, UDH, and FCC lesions showed peak currents lower than 196 μA. Hence, the responses of CDP are classifiable based on the newest edition of ductal intraepithelial neoplasia (DIN),^10^ lobular intraepithelial neoplasia,^11^ and fibro‐epithelial lesion^12^ systems reported by the WHO.^13^ Most abnormal samples were found in DIN classification (as the most occurred types of breast diseases such as IDC, DCIS, ADH, and UDH).

Nonmalignant and/or malignant lesions showed hypoxia‐related current peaks lower than 200 μA, and lesions involved to at least one focus of malignant or two foci of premalignant lesions showed current peaks more than 220 μA. So, an experimentally cut‐off was achieved for CDP responses, and a pathologically classified table includes negative and suspicious scores as noninvolved margins and positive scores as involved margins, which must be re‐excised, were defined in 203 μA (*p*‐value < 0.0001) (Figure 4, and Table S8). Such scoring would consider any pathological involvement to atypical, pre‐invasive, and invasive lesions in margin checking.

A warning regime in negative regions named as “Yellow Zone” was defined in this classification. These samples with current peaks between 137 and 196 μA are classified in this region. Surgeons need to be aware of these lesions through the presence of complex fibroadenoma, complex SA, or a single focus of atypia. Although these lesions are not pathologically high‐risk pre‐neoplasia, they should be reported and considered through some guidelines.^32^

This cut‐off definition's accuracy and specificity were 92% and 95%, respectively (Table S9).

**FIGURE 5:**
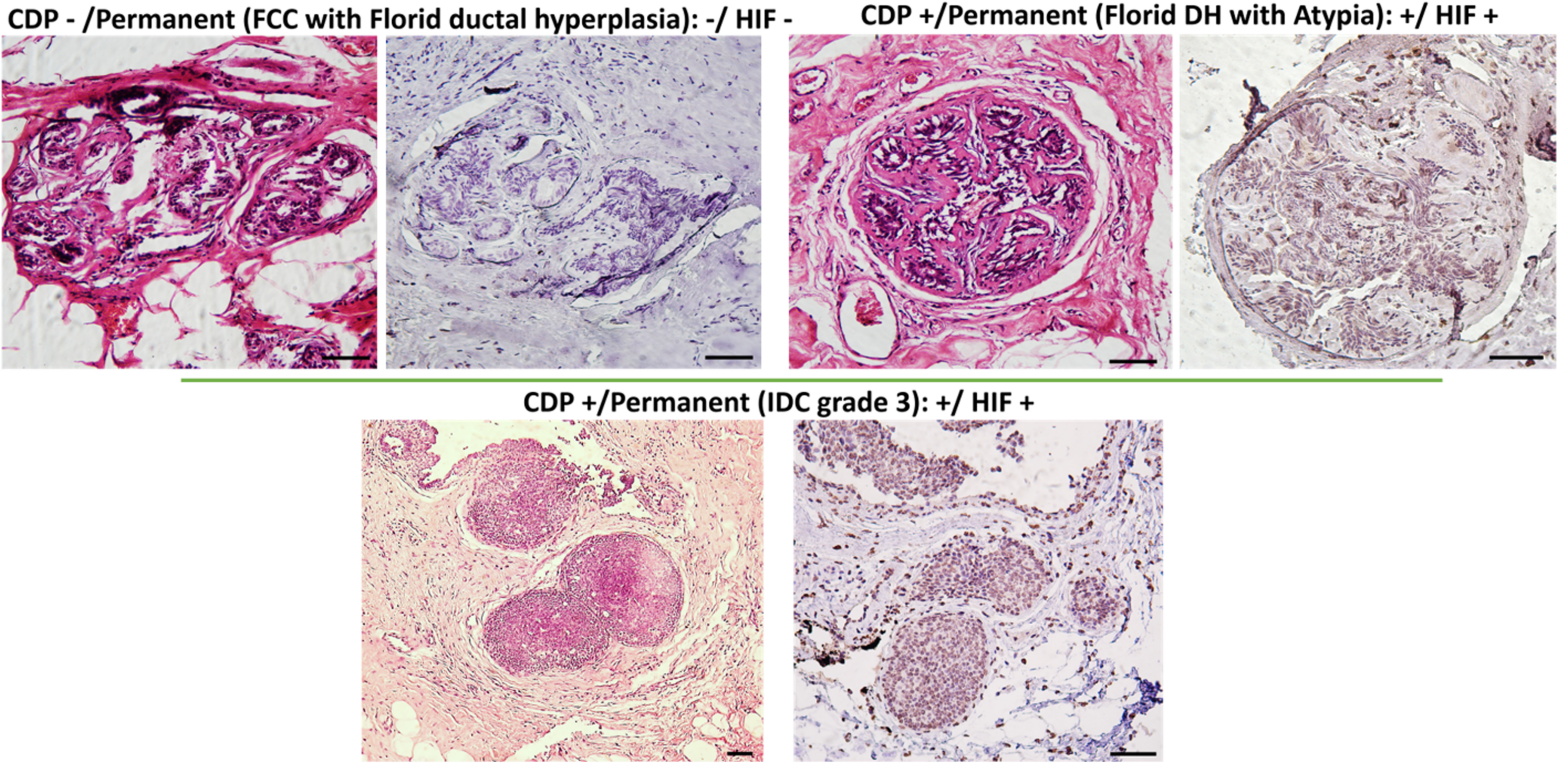
Further analyses by HIF1‐α IHC marker on some samples which CDP has negatively and positively scored. Neoplastic lesions showed expressed HIF‐1‐α with a positive CDP score, while benign lesions showed no expression of the hypoxia‐induced factor with a negative score of CDP

Moreover, our IHC evaluation from some lesions has been positively scored by CDP confirmed the expression of the hypoxia‐induced factor (HIF1‐α) marker. It is well known that expression of HIF‐1α shows a good correlation with hypoxia glycolysis and release of ROS/H_2_O_2_ by neoplastic cells.^33^, ^34^, ^35^ Further analyses by the HIF1‐α IHC marker showed that the breast samples which have been positively scored by CDP (such as atypia or invasive carcinoma) (Figure 5) expressed considerable levels of HIF1‐α. Moreover, no expression of HIF1‐α was observed on lesions with FCC and florid ductal hyperplasia, as benign non‐atypical samples). Those lesions had been truly scored negative by CDP. These results supported the specificity of CDP scoring based on the H_2_O_2_ released from neoplastic or atypical lesions through reverse Warburg effect,^5^, ^6^ and hypoxia assisted glycolysis pathways in a quantitative electrochemical manner.

## 3 DISCUSSION

CDP was introduced as a handheld real‐time diagnostic tool for detecting the lesions involved in atypical, pre‐neoplastic, and neoplastic breast cells with pathological classification capability. The detection has been carried out by electrochemical tracking of the metabolic pathways associated with tumor initiation, progression, and metastasis, such as reverse Warburg effects,^6^ hypoxia assisted glycolysis,^5^ and field‐effect.^36^ Quantitative scoring of pre‐invasive/invasive lesions in correlation with the standard pathological categorization of breast tumor diseases would be the distinct role of CDP in the future of cancer surgery. An increase in CDP peak currents through increased concentration or phenotypic progression of atypical/cancer lesions was meaningful. By in vitro and in vivo investigation of more than 250 tissue samples and 57 mice models, the best pathological scoring of CDP, based on WHO pathological classification of breast tumors, was experimentally proposed (with the sensitivity and specificity of 95% and 97%, respectively). The whole testing process on one sampling from preparing CDP to declaring the diagnosis takes place in less than 40 sec.

## 4 CONCLUSIONS

In summary, we developed a hypoxia‐based detection system to find cancerous breast lesions in real‐time. The system has been based on an electrochemical tracing of hypoxia glycolysis byproducts (H_2_O_2_/ROS) released from neoplastic cells in less than 40 sec by nanotextured needle electrodes. After confirming its capability on different types of human cell lines, the CDP system showed more than 95% matching with pathological results in testing more than 250 in vitro human fresh tumor samples and 57 in vivo tumorized mice models.

Finally, after passing many clinical trials and safety exams, CDP may be capable of receiving the clinical certificate as a useful method for margin diagnostics to cover the missed margins that might have remained during the BCS or partial mastectomy surgeries in the future. The capabilities of CDP seemed to be comparable with recently developed technologies such as MasSpec,^1^ margin probe,^2^ and CONVIVO^3^ (Tables S1 and S2). Pathologically classified responses on finding the involved lesions and margins, in their live states, ranged from pre‐invasive to invasive metastasized lesions, disposable head probe with the cheap fabrication process and no biological perturbation on the vitality and function of the tested lesions are the strengths of CDP.

## 5 MATERIALS AND METHODS

### 5.1 Fabrication of CDP for in vivo assays

First, the sterile needle was rinsed in deionized water and dried by air. A fixture was designed and fabricated to hold the needles both in E‐beam and DC‐PECVD systems to limit the growth of CNTs just in the tips of the needles. Ni catalyst layer for CNT growth with a thickness of 9 nm was coated on Ni needle by the E‐beam evaporation system (Veeco Co.), at the temperature of 120°C with a depositing rate of 0.1 Å/s. Afterward, Ni‐covered samples were located in a direct current plasma enhanced chemical vapor deposition (DC‐PECVD) system to grow vertically aligned multiwalled carbon nanotubes (VAMWCNTs). The growth has a three‐step process named annealing, graining, and growth. At first, the sample was annealed at 680°C in a H_2_ environment with a flow rate of 20 standard cubic centimeters per minute (sccm) for 30 min. During the graining, the surface was plasma hydrogenated for 5 min with the intensity of 5.5 W cm^−2^, which results in the catalyst graining and formation of Ni nano‐sized islands. In the growth step, plasma of C_2_H_2_ and H_2_ mixture with flow rates of 4.5 and 20 sccm was introduced to the chamber for 20 min. Finally, CNTs were characterized by FE‐SEM. The length and diameter of nanotubes ranged from 2.5 to 5 μm and from 50 to 70 nm, respectively. Figure 1b shows the FE‐SEM image of the CNT biosensor. These CNTs were MWCNTs of high purity, and the presence of nickel on the topside of the CNTs could be related to the tip‐growth mechanism. The CNT has been used as the work, counter, and reference electrodes. The CNT grown needles were then attached to an electrical connector with three pins by conductive paste to form the final probe. Just tips of the needle were extended from the connectors up to 1 cm. The probe was reinforced with a user‐friendly homemade holder and connected to the readout system by a noiseless cable that handled all three electrodes.

### 5.2 The CDP electrochemical readout system

The schematic of a CDP integrated portable automatic electrochemical readout board is shown in Figure S10. In this system, we used a low noise, high accuracy, and low power potentiostat. For making this potentiostat to decrease the noise loading effect in the environment caused by the other instruments in the operation room, we used low noise amplifiers. Moreover, to detect the current signal, which flows from the WE, a high‐speed potentiostat was required. So, the Current Buffer Amplifier Classes (CBAs) was added to the main board. These two classes of amplifiers produces a creative, effective, and appropriate device for high accuracy tests.

On the other hand, to increase the circuit's accuracy, analog to digital and digital to analog (DAC) converters with 16 bits was used. A processor unit is installed, which receives the converters' data and transmits them via a bluetooth module (Figure S10a).

Finally, the software was designed to analyze the data, diagnose cancerous or normal, and send the result through an alarm to the surgeon. A 3.3 V and 800 mA lithium‐ion cell battery is powering all parts of this circuit, so it does not require to the power supply. The device's ability provides a real‐time and accurate diagnostic method to utilize it clinically. The CV studies were done by DC voltage (no AC frequency) for electrochemical measurements. The potential was swept in the range from −800 to +800 m V, using a scan rate of 100 mV s^−1^ (Figure S10b).

### 5.3 Cell cultures and reagents

Breast cancer cell lines (MCF‐10A, MCF‐7, MDA‐MB‐231, MDA‐MB‐468), colon (COR‐L 105, SW‐480, HT‐29), hematopoietic (1301, LCL‐PI 1), liver (HEP G2), lung (QU‐DB, MRC‐5), mouth (KB), neuron (BE (2)‐C, LAN‐5), and prostate (PC‐3, Du‐145) were obtained from the standard cell banks of the National Cell Bank (NCBI) located in the Pasteur Institute. They were kept at 37°C (5% CO_2_, 95% air) in RPMI medium (Gibco) supplemented with 5% fetal bovine serum (Gibco) and 1% penicillin/streptomycin (Gibco). The fresh medium was renewed every other day. All cell lines were examined and found negative for *Mycoplasma* contamination and counted by neobar lam.

### 5.4 ROS assay

ROS generation was analyzed with a 5‐chloromethyl‐2′‐7′‐dichlorodihydrofluorescein diacetate (CM‐H2DCFDA) assay. This probe is changed to 2′–7′‐dichlorofluorescein (DCF) with a green fluorescent property by esterase enzymes in the cells' cytosol. After culturing the fibroblast and MCF‐7 cells overnight, the cells are washed with PBS, and then 500 μl of the CM‐H2DCFDA solution is added with a concentration of 20 μM. After 30 min of incubation at room temperature and dark, cells are again washed and then imaged with a fluorescent microscope. The cells were incubated with 6.5 mM of NAC as a ROS scavenger and then treated with CM‐H2DCFDA. Finally, the samples were imaged with a fluorescent microscopy system.^37^

### 5.5 l‐Lactate assay kit (colorimetric) procedure

The production of lactate in culture was analyzed to determine the rate of hypoxia‐assisted glycolysis. According to the manufacturer's protocol, the intracellular level of lactate was measured by the colorimetric lactate assay kit (Abcam: ab65331, UK). The optical densities were then measured at 450 nm wavelength. The assay was carried on due to the below steps:

1. *Reagent preparation*: Solubilize lactate substrate mix and lactate enzyme mix, thaw lactate standard, and lactate assay buffer (aliquot if necessary); get the equipment ready.
2. *Standard preparation*: Prepare lactate standard dilution (range 2–10 nmol/well) (according to the manufacturer's protocol).
3. *Sample preparation*: Prepare samples (including deproteinization step) in optimal dilutions to fit standard curve readings.
  - Cell (adherent or suspension) samples:
    - Harvest the number of cells necessary for each assay (initial recommendation = 2 × 10^6^ cells).
    - Wash cells with cold PBS.
    - Resuspend the cell pellet in 500 μl of lactate assay buffer.
    - Homogenize cells quickly by pipetting up and down a few times.
    - Centrifuge 2–5 min at 4°C at top speed in a cold microcentrifuge to remove any insoluble material.
    - Collect the supernatant and transfer it to a clean tube.
    - Keep on ice.
    - Cell samples include endogenous LDH which can degrade lactate. Remove enzyme from the sample by using deproteinizing sample preparation kit—TCA (ab204708). Alternatively, you may use a PCA/KOH deproteinization step due to the protocol described in section 11.4 in the kit instruction.
  - *Tissue samples*:
    - Derive cells from the tissue by applying collagenase.
    - Harvest the required quantity of cells for each assay (initial recommendation = 10 mg tissue).
    - Wash the sample by PBS in low temperature.
    - Re‐suspend cells in 4–6× volumes assay buffer with the assistance of a homogenizer (kept in ice), with 10–15 passes.
    - Centrifuge samples for 2–5 min at high speed at 4°C in a low temperature microcentrifuge to separate non‐soluble material.
    - Collect the supernatant and move it to another tube.
    - Keep on ice.
    - Samples include endogenous LDH with the ability of degrading the lactate. Enzyme would be removed from the sample by deproteinizing preparation kit—TCA (ab204708). Alternatively, you can use a PCA/KOH deproteinization procedure following the protocol described in section 11.4 in the instruction.
4. *Set up reaction wells*: Arrange a well in duplicate for standard, samples and background control all in volume of 50 μl.
5. *Reaction mix*: Supply a master mix for l‐lactate reaction mix and individual master mix for background reaction mix.
  - Add 50 μl of reaction mix to standard and sample wells followed by adding 50 μl background reaction mix to sample background control wells.
  - Keep plate at room temperature for 30 min.
  - Use LEISA reader for measuring plate at OD 450 nm in a microplate reader.

#### 5.5.1 Calculation

Duplicate the test for each standard and sample and extract the average. If the background control of the sample is significant, then deduct it from the sample readings. Decrease the mean absorbance value (Standard #1) from all standard and sample readings. Corrected absorbance will achieve. Consider the corrected absorbance values for each standard as a function of the final concentration of lactate.

To form the standard curve, draw the curve through these points. Most plate reader software can outline these values and curves. Based on your standard curve data, calculate the trend line equation (use the equation that presents the most accurate fit).

The concentration of l‐lactate in the test samples is determined as:

$$\text{Lactate concentration}=\left(\mathrm{La}/\mathrm{Sv}\right)*D,$$

La, amount of lactic acid in the sample well calculated from a standard curve (nmol); Sv, volume of sample added into the well (μl); *D*, sample dilution factor; lactic acid molecular weight, 90.08 g/mol.

### 5.6 Tumor formation in mice models

Sixty female inbred BALB/c mice at 6–8 weeks of age were acquired from Pasteur Institute of Iran. They were kept at 22–24°C with a 12 h light/dark cycle in a designed pathogen‐free isolation facility and allowed to adapt for a week before tests. The animal ethics committee approved all procedures. Animals were acclimated for 7 days before tumor cell injection and were housed individually in separate cages when not receiving the experimental treatments. They access to food (standardized mouse sterilizable diet) and water. In the next step, 3 × 10^6^ 4T1 cells/200 μl were injected into the back of the neck or right side. Tumor sizes were measured by portable sonography. At the time of the CDP test, the mice were about 8–10 weeks old and had body weights of 17 ± 0.4 and 19.8 ± 0.3 g, respectively (mean ± SEM). After 10 days, to assess the superficial tumor regions, individual head probes of CDP (fabricated with the distance of ~1 mm between the needles due to the mice's tumor sizes) were entered into them from the skin. All mice were anesthetized by IP injection with a combination of ketamine (80–100 mg/kg) and xylazine (10–12.5 mg/kg), which lasted about 20–30 min. During the test, normal body temperature was maintained using a warm circulating water blanket, thermal pads, and warmed IV fluids. The fluids (e.g., IV, IP, SQ) to animals during prolonged anesthesia to maintain adequate hydration was used. The ophthalmic ointment to both eyes was also applied to prevent desiccation for any anesthesia longer than 5 min. After surgery and removal of tumor margins for pathological examinations, to prevent infection and sepsis in anesthetized mice, retrobulbar injection of less than 200 μl of injectable anesthetic solution (ketamine: xylazine) were used to death within 5 s of cessation of injection.^38^

### 5.7 In vitro sample collection from the patients

All methods for the human subjects were also carried out under relevant guidelines and regulations. According to an ethically approved protocol (IR.TUMS.VCR.REC.1397.355) at our breast cancer central clinics and assistant hospitals, patients provided consent. Live slices from CNB or surgically removed samples were cut into similar specimens and directly transferred through microwells containing RPMI‐1640 without preprocessing (Table S11). To better clarify the ability of CDP as a real‐time detection system, two movies (under the ethics committee ID: IR.TUMS.VCR.REC.1397.355 ) were presented in supplementary (Movie S1, S2).

### 5.8 Tissue staining procedure with H&E

H&E is used to illustrate the nucleus and cytoplasmic inclusions in clinical specimens. Hematoxylin works as a mordant and stains the nucleus light blue. In the presence of an acid, the dye turns red. To achieve differentiation, we should put the tissue in an acid solution. By using eosin, the counterstaining is done, which provides pink color to the cytoplasm. The H&E staining process starts with a tissue section's deparaffinization, flaming the slide on the burner, and placing it in the xylene. After the hydration process, the treatment process must be repeated. To hydrate the tissue section, it should pass through a decreasing concentration of alcohol (100%, 90%, 80%, and 70%). Next, for approximately 3–5 min, the sample should be stained in hematoxylin, followed by washing in the tap water until the sections become blue. The sample should be contrasted in 1% acid alcohol (1% HCl in 70% alcohol) for 5 min in the next step. Afterward, the sample should be washed in running tap water, soaked in an alkaline solution (e.g., ammonia water) until it turns blue, followed by another tap water wash. Moreover, the sample should be stained in 1% eosin Y for 10 min and washed in tap water for 1–5 min. Finally, the sample should be soaked in an increasing concentration of alcohol to dehydration and then clear within the xylene.

### 5.9 Patients' samples staining by the IHC procedure

Discrepancy cases were picked for further IHC procedures. The samples were cut and put‐on poly l‐lysine coated slides. Using a microwave oven with heat‐induced epitope retrieval antigen, retrieval was conducted. By the clone AE1/AE3 (dilution 1:50), avidin‐biotin method (utilizing labeled streptavidin‐biotin [LSAB] + kit) with Dako monoclonal anti‐human PCK, anti‐HIF‐1α antibody (ab82832), anti‐cytokeratin 5 + 6 antibody [D5/16 B4] (ab17133), anti‐cytokeratin 14 antibody [LL002] (ab7800), and recombinant anti‐p63 antibody [EPR5701] (ab124762) the IHC were done.

### 5.10 RNA sequencing of breast cell lines

RNA samples, including MCF‐10A, MCF‐7, MDA‐MB 231, and MDA‐MB 468, are extracted from the breast cell lines, thawed and incubated for 90 s at 70°C. To produce cDNA, we treated samples with reverse transcription master mix, incubated them on a thermocycler at 50°C for 30 min, and 70°C for 15 min. To extract the free primers, we added 1.0 μl of EXOSAP mix to each sample, then incubated the mixture at 37°C for 30 min, and inactivated it at 80°C for 25 min. Afterward, by incubating in the master mix at 37°C for 15 min, and inactivated at 70°C for 10 min, a 3′ poly (A) tail was added to the cDNA in each sample. By dividing each sample into four and incubating in master mix at 95°C for 3 min, 50°C for 2 min, and 72°C for 10 min, the second layer of cDNA was integrated. PCR increase (95°C for 3 min, and then, 20 cycles of 95°C for 30 s, 67°C for 1 min, and 72°C for 6 s) was performed with the master mix. Each sample's four responses were pooled, filtered applying the Qiagen PCR purification kit (cat. no. 28106), and eluted in 50 μl EB buffer (cat. no. 28106), and eluted in 50 μl EB buffer. By testing genes, HIF‐1α, c‐Myc, HKII, PGAM1, and LDHA were chosen.

Primers and probes (Table S12) were produced by AlleleID (Premier Biosoft) and synthesized by BonYakteh Corp. Nucleotide series applied for the probe‐primers design were obtained from the NCBI database. BLAS aligned the designed probe‐primers to approve gene specificity. Again, each sample was divided into four, and the second round of PCR amplification (nine cycles of 98°C for 3 min, 67°C for 1 min, and 72°C for 6 min 6 s) was made with a master mix. Samples were pooled and washed using Agencourt AMPure XP beads and eluted in 40 μl 1× low‐TE buffer (Table S12).

### 5.11 Statistical analysis

SPSS software (version 26) was used for statistical analysis. To evaluate each of the diagnostic tests (CDP and frozen conventional pathology), the ROC curves and AUC have been done to assess and compare the detection ability of each group with the gold standard test (permanent pathology). Also, the sensitivity, selectivity, accuracy, and specificity of each of them were calculated with SPSS software. A *p*‐value of less than 0.01 was considered significant.

## CONFLICT OF INTEREST

Four USA patents (one granted; US Patent US10,786,188 B1, and three publications; US Patent App. US2018/0299401 A1, US2021/0007638 A1, and US2021/0022650 A1) have been published based on this work. M.A. is a member of the scientific advisory board of Arya Nano biosensor Manufacturer Co., a company that is commercializing CDP technology. The remaining authors declare that they have no competing interests.

## AUTHOR CONTRIBUTIONS

**Zohreh Sadat Miripour:** Data curation; formal analysis; investigation; methodology. **Fereshteh Abbasvandi:** Investigation; validation; visualization. **Parisa aghaee:** Formal analysis; investigation; visualization. **Sahar NajafiKhoshnoo:** Investigation; visualization. **Mahsa Faramarzpour:** Investigation; visualization. **Pooneh Mohaghegh:** Investigation; visualization. **Parisa Hoseinpour:** Investigation; validation; visualization. **Naser Namdar:** Methodology; software. **Morteza Hassanpour Amiri:** Methodology; software. **Hadi Ghafari:** Methodology; visualization. **Fatemeh Shojaeian:** Investigation; visualization. **Hassan Sanati:** Investigation; visualization. **Mahna Mapar:** Investigation; visualization. **Nastaran Sadeghian:** Investigation; validation; visualization. **Mohammad Esmaeil Akbari:** Methodology; validation. **Mohammad Ali Khayamian:** Investigation. **Mohamad Abdolahad:** Investigation, Conceptualization; project administration; supervision; writing‐original draft; writing‐review & editing.

## Supporting information


**Table S1.** Specifications of various margin detection technologies
**Table S2**. Comparative capabilities of recently published margin detection technologies.
**Table S3**. Comparative table of CDP and H&E results of the tumor margins (R1: center of the tumor, R2: right, R3: left, R4: up, and R5: bottom margins of the tumor. R6 is a normal region far from the tumor) in 57 mice had been tumorized by the injection of 4 T1 cell lines. The tested margins were up to 1 mm outer from the visible boundaries of the tumor
**Table S4**. CDP scores vs. Permanent Cross tabulation for 57 in‐vivo mice samples
**Table S5**. FROZEN * PERMANENT Cross tabulation for 57 in‐vivo mice samples
**Table S6**. Area Under the Curve (AUC) for CDP vs. permanent results in 57 in‐vivo mice samples
**Table S7**. AUC table for frozen vs. permanent results in 57 in‐vivo mice samples
**Table S8**. Examples of DIN‐CDP scored in‐vitro samples of dissected tissues through surgery or biopsy from 74 breast disease patients proposed based on classification introduced in Figure 4.
**Table S9**. Cross tabulation results for CDP vs. permanent as a gold standard for 258 in‐vitro samples from 74 patients.
**Table S10**. AUC for CDP results vs. permanent for 258 in‐vitro samples from 74 patients
**Table S11**. Quick‐Reference BRISQ Summary/Checklist.
**Table S12**. Oligonucleotide primer and probe sequences used in the present study.
**Figure S1**. a) The effect of needle sterilization on the electrochemical cathodic peak. b) The SEM image of biological foam cross‐section after injection of a needle for the first time, b1‐b2) Higher resolution SEM from the remained residue of CNT on the sponge c‐d) The SEM image of a cross‐section of individual biological sponge, after 3rd injection of the same CDP needle. Therefore, weakly bonded CNTs were released from the needle (after three times injection into the sponge), and strongly bonded ones would not remain any residue on a sponge.
**Figure S2**. CDP responses on tumor and normal fresh breast tissue recorded by a) all CNT covered, b) WE covered by CNT, and c) non‐CNT covered head probes. All CNT covered electrodes achieved best‐distinguished responses. d) FE‐SEM image of the CNT grown on needle electrodes after squeeze to the fresh breast tissue and rinsing in Ethanol. No considerable perturbation of destruction was observed in post‐squeezed probes.
**Figure S3**. Repeatability test on some randomly selected the fabricated CDP head probe (vertical axis: number of the fabricated probe). Well acceptable similarity in responses was observed for head probes.
**Figure S4**. Effect of CNT on the viability of MCF‐10A (breast cancer cells) and HUVEC (vein endothelial cells) at 24 and 72 hours of incubation
**Figure S5**. a) solution media's CV responses of different normal and cancerous cell lines in various phenotypes. The current peaks in the cancerous samples were observably increased. The lactate based oxidative peaks of cancer media solutions were sharper than that in normal cells. We observed grade dependent increase in lactate peaks of cancer cells in progressive phenotypes, and b) A comparative columnar diagrams of the CV responses of different cell lines.
**Figure S6**. Top: Tumorized mouse by 4 T1 breast cancer cell lines revealed by sonography and H&E images taken before and after testing by CDP, respectively. Bottom: CV diagrams of normal and tumor regions calibrated by lactate 0.3 mM. The lactate related peaks were about three times higher in the cancer region vs. normal ones.
**Figure S7**. Receiver Operating Characteristic (ROC) diagram for CDP vs. permanent results in 57 in‐vivo mice samples
**Figure S8**. ROC diagram for frozen vs. permanent results in in 57 in‐vivo mice samples
**Figure S9**. ROC diagram for CDP vs. permanent results in 258 in‐vitro samples from 74 patients
**Figure S10**. The schematic of an integrated portable automatic electrochemical readout board. Bottom panel: Schematic of the calculating procedure of CDP response value.

**Movie S1** Supporting information.

**Movie S2** Supporting information.

## Data Availability

Our data is available upon request of the Editor.
