# Supplementary material for: Electrochemical tracing of hypoxia glycolysis by carbon nanotube sensors, a new hallmark for intraoperative detection of suspicious margins to breast neoplasia
Source: Bioeng Transl Med. 2021 Jun 14;7(1):e10236. doi: 10.1002/btm2.10236 (PMC8780057; doi:10.1002/btm2.10236)
Supplement: Supplementary file 1 — Table S1. Specifications of various margin detection technologies Table S2. Comparative capabilities of recently published margin detection technologies. Table S3. Comparative table of CDP and H&E results of the tumor margins (R1: center of the tumor, R2: right, R3: left, R4: up, and R5: bottom margins of the tumor. R6 is a normal region far from the tumor) in 57 mice had been tumorized by the injection of 4 T1 cell lines. The tested margins were up to 1 mm outer from the visible boundaries of the tumor Table S4. CDP scores vs. Permanent Cross tabulation for 57 in‐vivo mice samples Table S5. FROZEN * PERMANENT Cross tabulation for 57 in‐vivo mice samples Table S6. Area Under the Curve (AUC) for CDP vs. permanent results in 57 in‐vivo mice samples Table S7. AUC table for frozen vs. permanent results in 57 in‐vivo mice samples Table S8. Examples of DIN‐CDP scored in‐vitro samples of dissected tissues through surgery or biopsy from 74 breast disease patients proposed based on classification introduced in Figure 4. Table S9. Cross tabulation results for CDP vs. permanent as a gold standard for 258 in‐vitro samples from 74 patients. Table S10. AUC for CDP results vs. permanent for 258 in‐vitro samples from 74 patients Table S11. Quick‐Reference BRISQ Summary/Checklist. Table S12. Oligonucleotide primer and probe sequences used in the present study. Figure S1. a) The effect of needle sterilization on the electrochemical cathodic peak. b) The SEM image of biological foam cross‐section after injection of a needle for the first time, b1‐b2) Higher resolution SEM from the remained residue of CNT on the sponge c‐d) The SEM image of a cross‐section of individual biological sponge, after 3rd injection of the same CDP needle. Therefore, weakly bonded CNTs were released from the needle (after three times injection into the sponge), and strongly bonded ones would not remain any residue on a sponge. Figure S2. CDP responses on tumor and normal fresh breast tissue recorded by a) all [file BTM2-7-e10236-s001.pdf]

Supplementary

Electrochemical tracing of Hypoxia glycolysis by carbon nanotube sensors, a new hallmark for real-time detection of suspicious lesions to breast neoplasia

Zohreh Sadat Miripour[1,2,#], Fereshteh Abbasvandi[3,$], Parisa Aghaee[1,2,$], Sahar NajafiKhoshnoo[1,$], Mahsa Faramarzpour[1,2,$], Pooneh Mohaghegh[1,2,$], Parisa Hoseinpour[5,$], Naser Namdar[1,2], Morteza Hassanpour Amiri[1,2], Hadi Ghafari[1], Sarah Zareie[1,2], Fatemeh Shojaeian[4] , Hassan Sanati[6], Mahna Mapar[1], Nastaran Sadeghian[3], Mohammad Esmaeil Akbari[4], Mohammad Ali Khayamian[1,2] and Mohammad Abdolahad[1,2,7,8 #,*]

1. Nano Bio Electronic Devices Lab, School of Electrical and Computer Engineering, College of Engineering, University of Tehran, P.O. Box 14395/515, Tehran, Iran

2. Nano Electronic Center of Excellence, Thin Film and Nanoelectronics Lab, School of Electrical and Computer Engineering, College of Engineering, University of Tehran, P.O. Box 14395/515, Tehran, Iran

3. ATMP Department, Breast Cancer Research Center, Motamed Cancer Institute, ACECR, P. O. Box 1615179/64311 Tehran, Iran

4. SEPAS Pathology Laboratory, P.O. Box: 1991945391, Tehran, Iran.

5. Cancer Research Center, Shahid Beheshti University of Medical Sciences, 198396-3113, Tehran, Iran

6. Integrative Oncology Department, Breast Cancer Research Center, Motamed Cancer Institute, ACECR, 19 P.O. Box 15179/64311, Tehran, Iran

7. Cancer Institute, Imam Khomeini Hospital, Tehran University of Medical Sciences, P.O. Box: 13145-158, Tehran, Iran.

8. UT&TUMS Cancer electro techniques Research Center, YAS Hospital, Tehran University of Medical Sciences, P.O. Box: 1598718311, Tehran, Iran.

*Corresponding Author: m.abdolahad@ut.ac.ir, abdolahad@tums.ac.ir

#,$ Authors with same contributions

Table sup1. Specifications of various margin detection technologies

Method [Ref.]	Mechanism	Tested Disease	Gold Standard	Declared Accuracy	Declared Strength	# of Tested Samples
**Mass Spec Pen** [1]	Controlled pure water droplet delivered to sampled tissue and extracted to mass spectrometer to analyze molecules according to their mass to charge ratio in proportion to their abundance	Lung Cancer, Breast Ductal Carcinoma, Papillary Thyroid Carcinoma, FTA	Pathology H&E Stained,	96.4% Sensitivity<br>96.2% Specificity<br>96.3% Overall accuracy	Automated, biocompatible, Disposable handheld device, Nondestructive sampling, Real time molecular diagnosis of tissue, Prediction of cancer	253 patient's ex vivo, 20 thin tissue section of human breast tissue (Normal and Ductal Carcinoma) and Thyroid Tissue (PTC & FTA)
**Brain cancer detection with RAMAN** [2]	A 785 nm NIR laser connected to fiber optics cable with a high-resolution CCD detector used with RAMAN fiber optic probe to detect invasive brain cancer in situ intraoperatively	Brain Cancer Grade 2 to 4 Gliomas	MRI, Pathology H&E	> 90%	Real time- High accuracy, Handheld small probe, Rapidly detection during surgery, Highly sensitive and specific to brain cancer tissue	161 Measurement in 17 patient undergoing brain cancer surgery
**Confocal endomicroscopy (CONVIVO)** [3]	A miniaturized confocal laser-scanning microscope has been developed in a hand-held probe and can be in a direct contact with patient's tissue to create a histology -like image. A 488 nm laser emission pass through fiber cable and excite the fluorescent dye injected intravenously which can be detected and interpreted as an image	brain surgery-tumor histology, including: gliomas, meningiomas, hemangioblastomas, central neurocytomas	permanent H&E stained pathology	> 90%	intraoperative detection of abnormal tissue, complementation of frozen section, real time biopsy (or digital biopsy), pathologist remote access to the confocal image through a connected network	mice and human ex vivo samples

**Margin probe** [4]	Utilize electromagnetic waves to characterize human tissue in real time. using radio frequency spectroscopy technology based on different responses reflected from cancerous and normal cells to electromagnetic waves	breast cancer		> 70%	reduce the rate of additional surgery on a lumpectomy, short time response, user friendly application	600 human patients
**CDP**	Electrochemical measurement of H_2O_2 release as a byproduct in hypoxia assisted glycolysis reaction activated in tumor cells with CNT covered electrodes	Breast Cancer	Frozen, Permanent Pathology and IHC	95% sensitivity<br><br>97% specificity	Additive ability to check the internal margins, Simple handheld probe, Precise, real time and fast response	on cell lines, in-vivo mice models with breast cancer and in-vitro fresh samples of breast cancer tumors

Table sup2. Comparative capabilities of recently published margin detection technologies

**Method [Ref.]**	**Declared detection of pre-neoplastic cells for each type of disease**	**Detection of neoplastic cells**	**Human in-*vivo* clinical investigation of internal margins**	**Animal model test**	**Pathological Cut off Differentiating between normal/ low risk benign/ High risk benign /cancer cells**	**types of disease tested *in-vitro***
Mass Spec Pen [1]	ADH ✗<br>Low garde DCIS ✗<br>SA ✗<br>Complex Fibroadenoma ✗	✓	✗	✓	✗	Lung Cancer, Breast Ductal Carcinoma, Papillary Thyroid Carcinoma, FTA
Margin probe [4]	ADH ✗<br>DCIS ✓<br>SA ✗<br>Complex Fibroadenoma ✗	✓	✓	✗	✗	breast cancer

Matrix-assisted Laser Desorption/Ionization Time-of-Flight Mass Spectra from Laser Capture Microdissected Tissues [5]	ADH ✗<br>Low garde DCIS ✓<br>SA ✗<br>Complex Fibroadenoma ✗	✓	✗	✗	✗	breast cancer, Lymph Node Metastasis
CDP	ADH ✓<br>Low garde DCIS ✓<br>SA ✓<br>Complex Fibroadenoma ✓	✓	✓	✓	✓ (more than one focus of ADH in $3mm^2$)	Breast Cancer

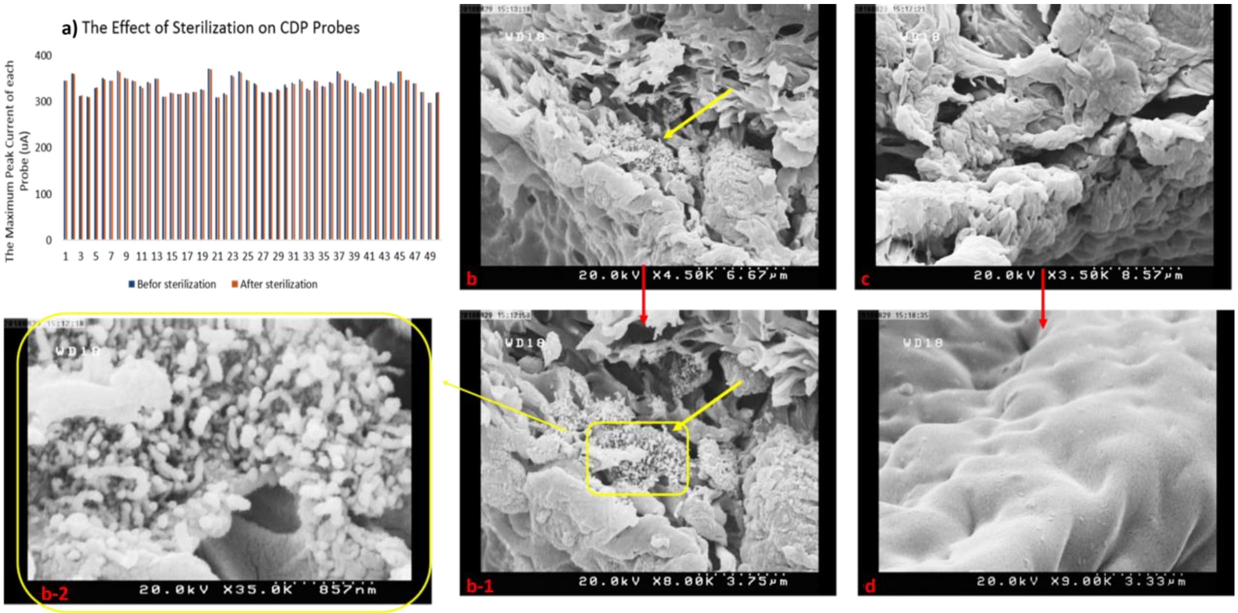

Figure Sup1. a) The effect of needle sterilization on the electrochemical cathodic peak. b) The SEM image of biological foam cross-section after injection of a needle for the first time, b1-b2) Higher resolution SEM from the remained residue of CNT on the sponge c-d) The SEM image of a cross-section of individual biological sponge, after 3rd injection of the same CDP needle. Therefore, weakly bonded CNTs were released from the needle (after three times injection into the sponge), and strongly bonded ones would not remain any residue on a sponge.

Also previous reports indicated on the application of such CNTs in selective electrochemical tracing of H_2O_2 [6],[7]. In the case of electrical conductivity and sharp signal extraction, we can

observe that presence of CNT on the surface of all involving electrodes strongly increased the intensity and symmetry of the responses (Supplementary Fig.3). This revealed the important role of CNT in selective interaction and charge transfer from the H_2O_2 released during hypoxic metabolism of neoplastic lesions. Presence of CNTs increased the interactive sites to more than thousands of times as could be observed in field emission scanning electron microscopy (FESEM) image (Supplementary Fig.3d). Also entrance of the needles into the tissue did not disturb the morphology of nanotubes (Supplementary Fig.3d). The production process of the CNTs has been stablished (IMED national ID: 14006918495) and is cost effective for us.

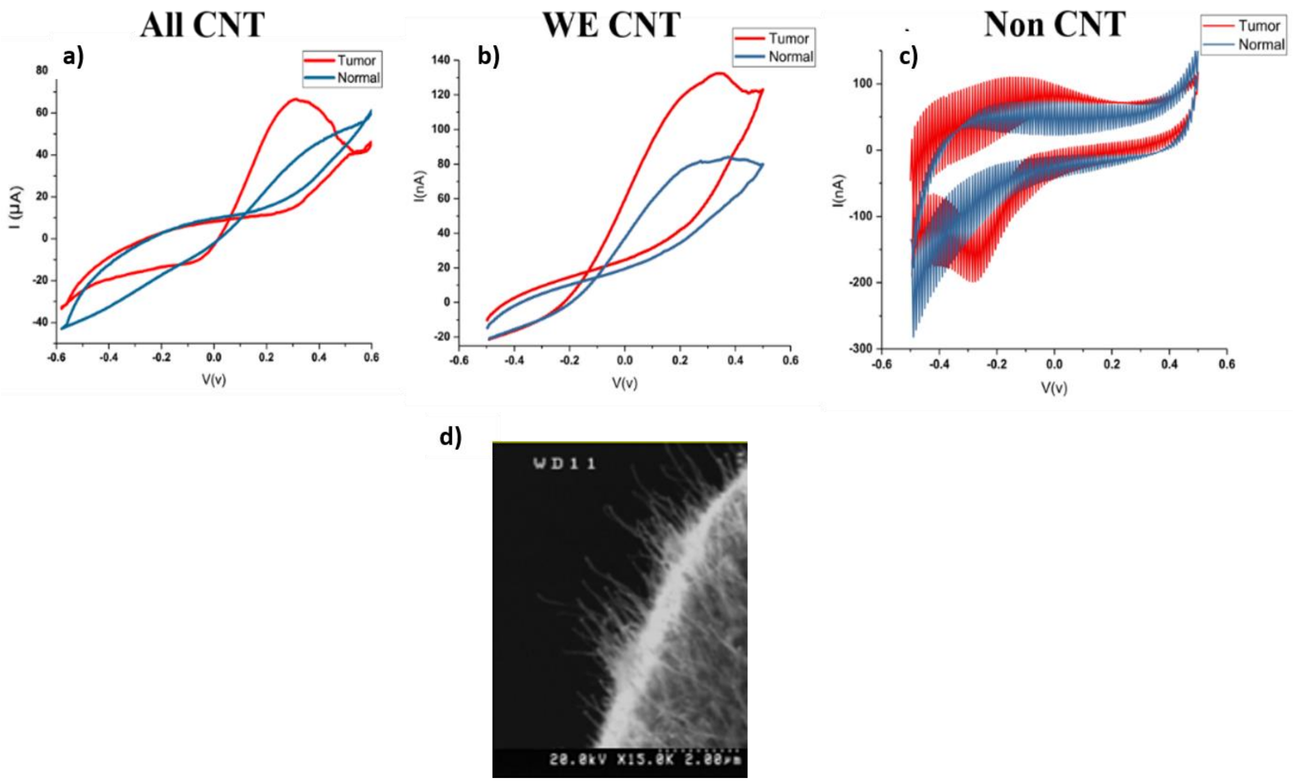

Figure Sup2. CDP responses on tumor and normal fresh breast tissue recorded by a) all CNT covered, b) WE covered by CNT, and c) non-CNT covered head probes. All CNT covered electrodes achieved best-distinguished responses. d) FE-SEM image of the CNT grown on needle electrodes after squeeze to the fresh breast tissue and rinsing in Ethanol. No considerable perturbation of destruction was observed in post-squeezed probes.

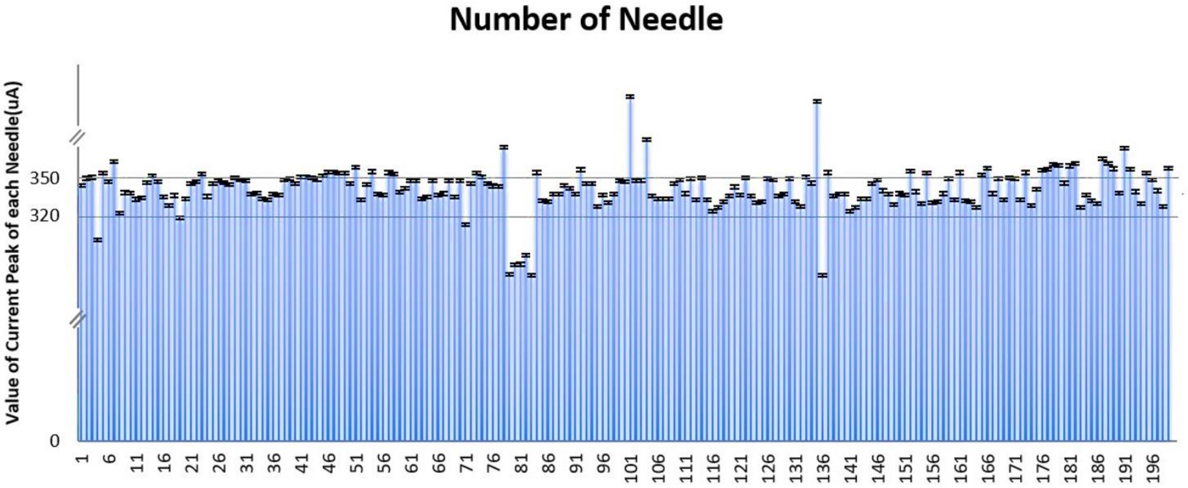

Figure Sup3. Repeatability test on some randomly selected the fabricated CDP head probe (vertical axis: number of the fabricated probe). Well acceptable similarity in responses was observed for head probes.

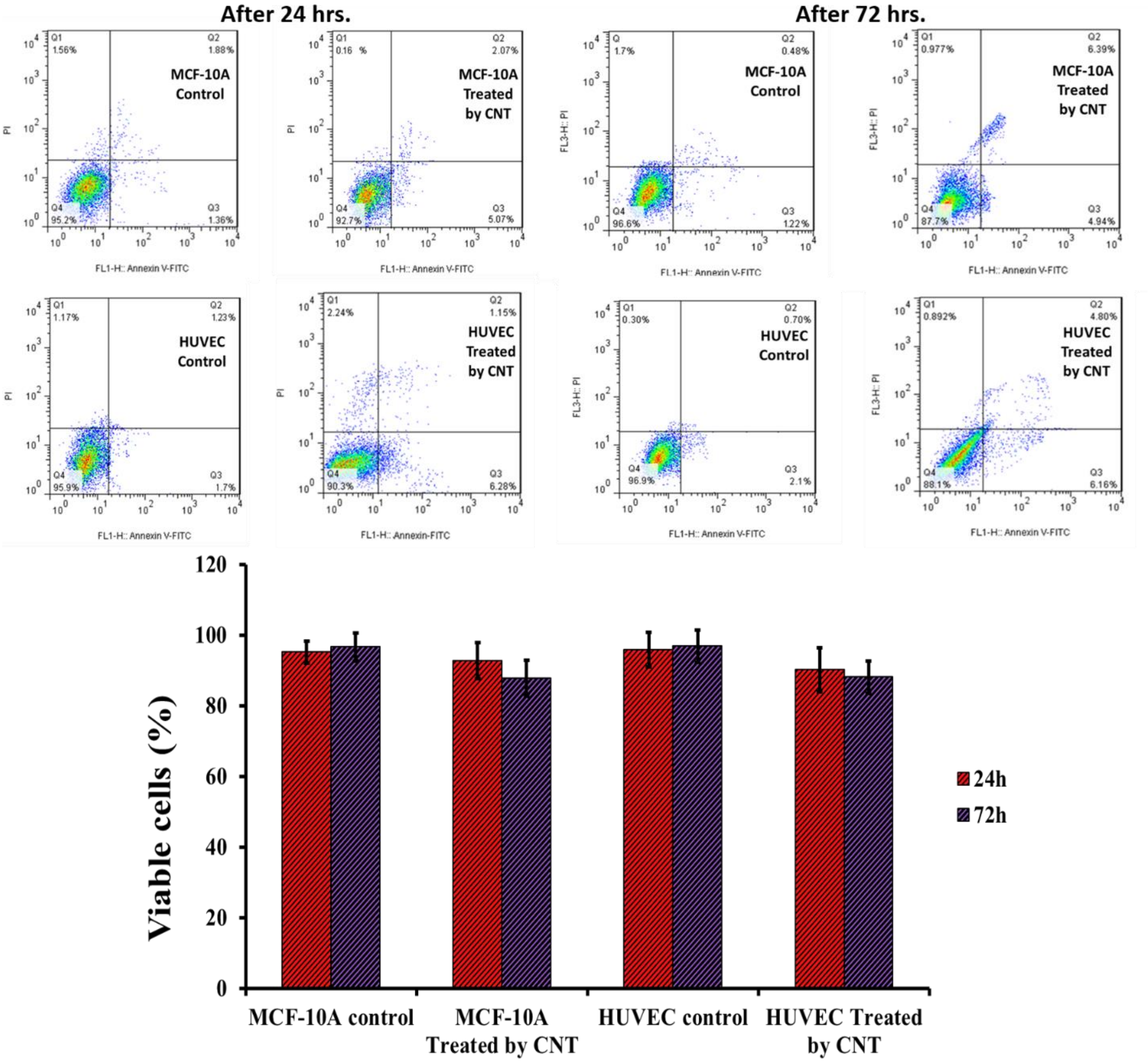

Figure Sup4 . Effect of CNT on the viability of MCF-10A (breast cancer cells) and HUVEC (vein endothelial cells) at 24 and 72 hours of incubation

S1. Cell cultures and reagents

Breast cancer cell lines (MCF10A, MCF-7, MDA-MB-231, MDA-MB-468), Colon (COR-L 105, SW-480, HT-29), Hematopoietic (1301, LCL-PI 1), Liver (HEP G2), Lung (QU-DB, MRC-5), Mouth (KB), Neuron (BE (2)-C, LAN-5), Prostate (PC-3, Du-145) were obtained from the standard cell banks of the National cell bank (NCBI) located in the Pasteur institute. They were

maintained at 37°C (5% CO_2, 95% air) in RPMI medium (Gibco) supplemented with 5% fetal bovine serum (Gibco), and 1% penicillin/streptomycin (Gibco). The fresh medium was replaced every other day. All cell lines were tested and found negative for Mycoplasma contamination. The cells were detached from the plates by trypsin and counted by neobar laam.

(a)

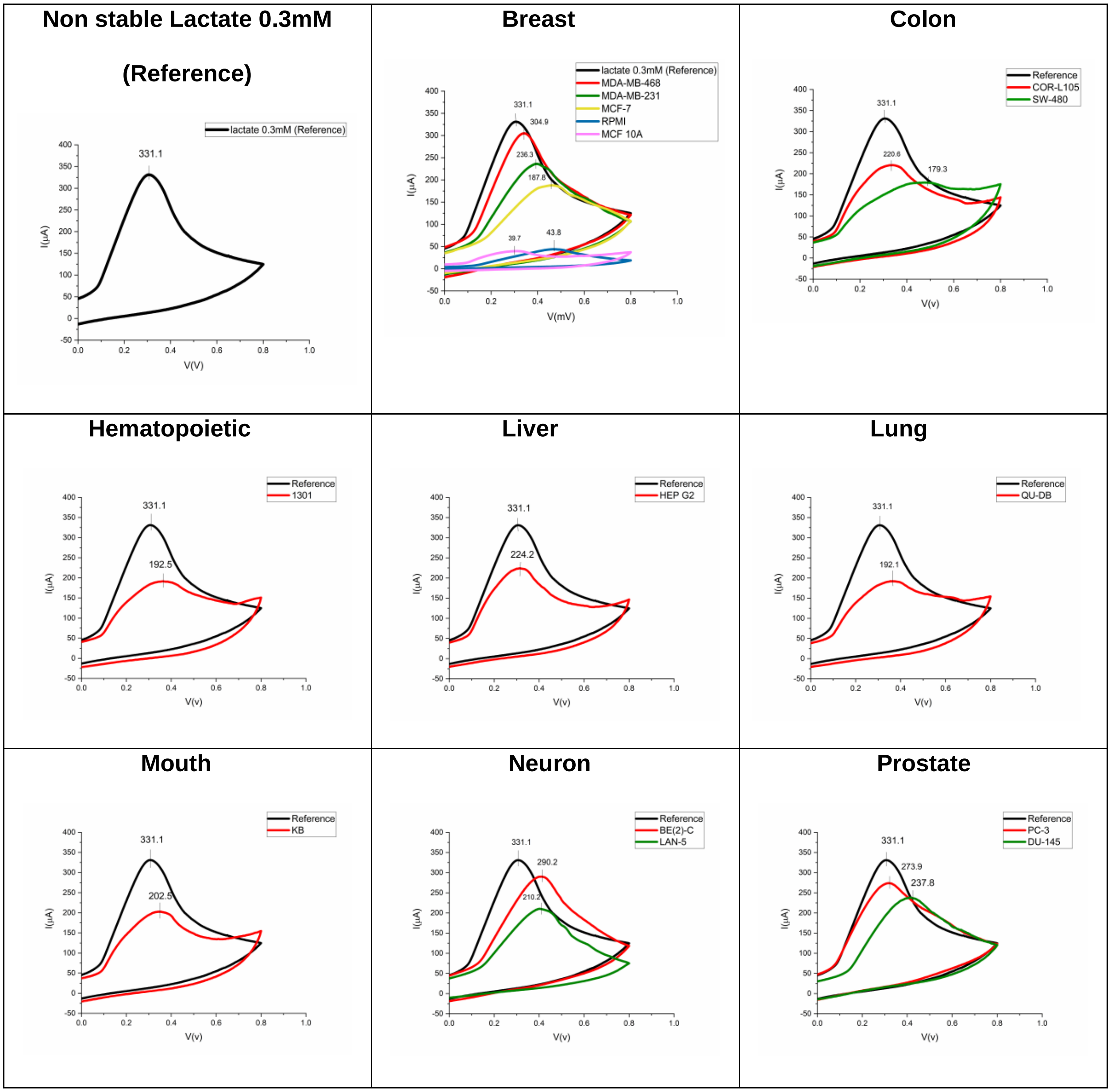

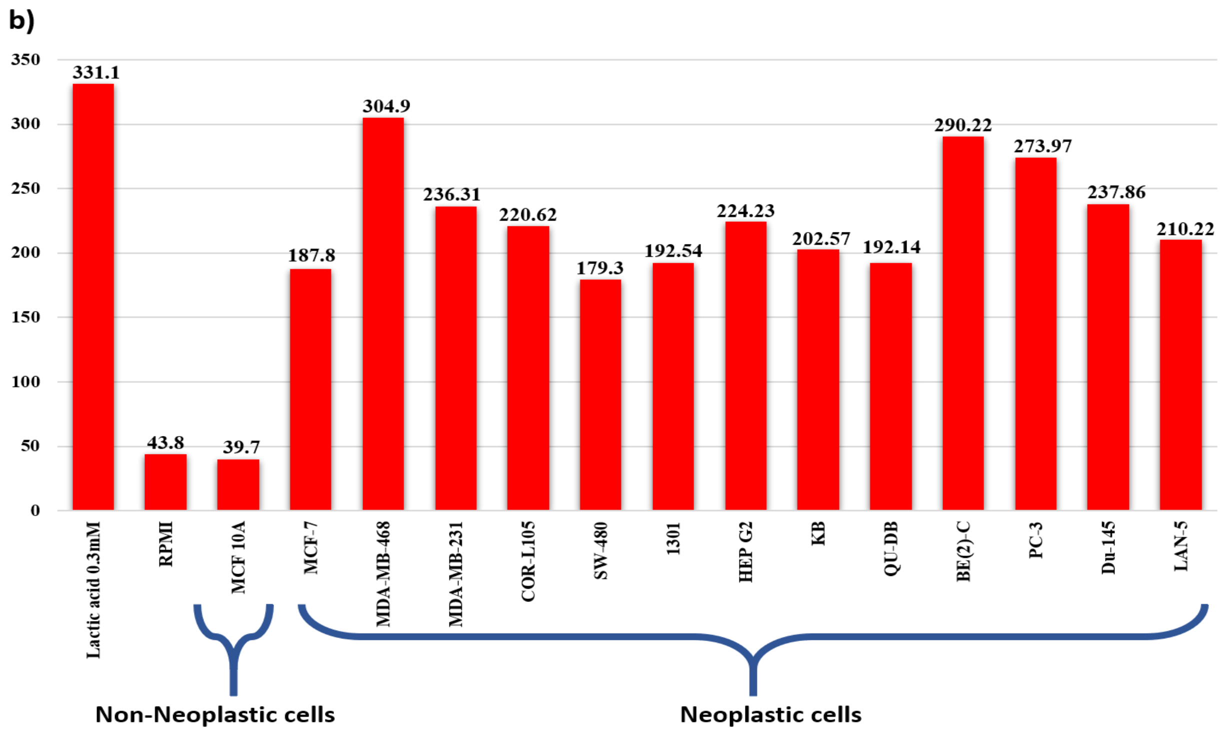

Figure Sup5. a) solution media's CV responses of different normal and cancerous cell lines in various phenotypes. The current peaks in the cancerous samples were observably increased. The lactate based oxidative peaks of cancer media solutions were sharper than that in normal cells. We observed grade dependent increase in lactate peaks of cancer cells in progressive phenotypes, and b) A comparative columnar diagrams of the CV responses of different cell lines.

S2. *In-vivo* studies of CDP in animal models

S2.1. Tumor formation in animal models

Female inbred BALB/c mice at 6–8 weeks of age were purchased from Pasteur Institute of Iran. They were kept at 22–24 °C with a 12 h light/dark cycle in an utterly designed pathogen-free isolation facility and allowed to adapt for one week before experimentation. Our animal ethics committee approved all procedures. A total of 3×10^6 4T1 cells/200µl in the logarithmic growth phase were subcutaneously (s.c.) injected into the back neck or right side of BALB/c mice. Tumor size was measured using a portable sonogram. In the case of non-malignant tumor injection, a total

of 3 $\times 10^6$ MC4L2 cells/200µl in the logarithmic growth phase were subcutaneously (s.c.) injected into the right side of BALB/c mice.

S2.2. Procedures of Mice tumorizing and CDP testing

We implanted 2.3×10^6 4T1-derived cancer cells (mouse type of invasive breast cancer cells) into the back of 60 female BALB/C mice. We maintained them in individual groups with similar sizes of formed tumors with sharp histological distinct patterns. After ten days, individual head probes of CDP (fabricated with a distance of ~1mm between the needles due to small tumor sizes in the mice) were externally entered from the skin to superficial tumor regions.

The distance between each assayed region was about 1 mm as the minimum distinguishable regions of CDP. A closer distance between the needle electrodes would induce some electrical noises and perturb the response. The mice underbody is connected to ground potential such as done for any patient in the surgery room.

Sharp electrochemical peaks were observed in tumor locations, about three times higher than the current recorded from their normal regions. Moreover, histopathological images taken from the normal and cancer regions detected by CDP confirmed this result. Hyper chromatic and irregular nucleus with an increased nucleus/cytoplasm ratio is observable in H&E images of the cancer region [8]. Moreover, the current peaks recorded from high-grade metastatic tumors (4T1) was higher than tumors with lower grades (MC4L2) (Supplementary Fig.6).

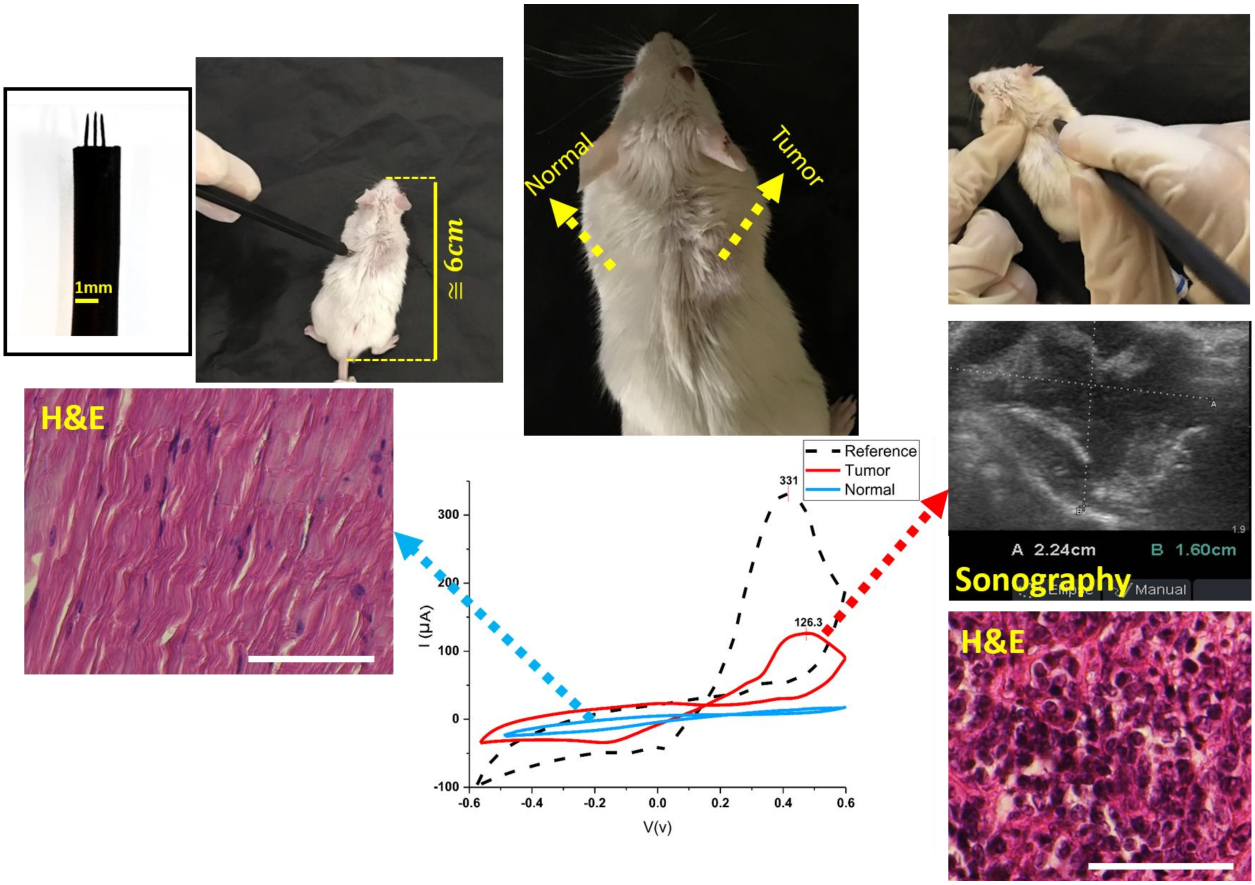

Figure Sup6. Top: Tumorized mouse by 4T1 breast cancer cell lines revealed by sonography and H&E images taken before and after testing by CDP, respectively. Bottom: CV diagrams of normal and tumor regions calibrated by lactate 0.3mM. The lactate related peaks were about three times higher in the cancer region vs. normal ones.

Table sup3. Comparative table of CDP and H&E results of the tumor margins (R1: center of the tumor, R2: right, R3: left, R4: up, and R5: bottom margins of the tumor. R6 is a normal region far from the tumor) in 57 mice had been tumorized by the injection of 4T1 cell lines. The tested margins were up to 1mm outer from the visible boundaries of the tumor.

Red: positive margins; dissection is mandatory.

Green: negative margins; dissection is not recommended.

(+): involved margins to cancer cells.

(-): free margins from cancer cells

CDP false positive: mouse's ID 52,53 and 55)

Mouse ID	R1			R2			R3			R4			R5			R6		
	CDP peak current	Frozen	Permanent	CDP peak current	Frozen	Permanent	CDP peak current	Frozen	Permanent	CDP peak current	Frozen	Permanent	CDP peak current	Frozen	Permanent	CDP peak current	Frozen	Permanent
1	234.45	+	+	0	-	-	193.5	+	+	8	-	-	26.05	-	-	31.26	-	-
2	312.6	+	+	38	-	-	0	-	-	0	-	-	198.7	+	+	63	-	-
3	383.6	+	+	25	-	-	199.6	+	+	0	-	-	380.3	+	+	10	-	-
4	292	+	+	0	-	-	221	+	+	16	-	-	255	+	+	52.1	-	-
5	295.6	+	+	12	-	-	110	-	-	26.05	-	-	0	-	-	0	-	-
6	225.8	+	+	0	-	-	192	+	+	1.5	-	-	0	-	-	0	-	-
7	420	+	+	125	-	-	78	-	-	45	-	-	295.6	+	+	13.9	-	-
8	356	+	+	267.5	+	+	321.8	+	+	57.9	-	-	26.7	-	-	0	-	-
9	337	+	+	0	-	-	11	-	-	0	-	-	193.5	+	+	47.5	-	-
10	358	+	+	26.05	-	-	190.5	+	+	0	-	-	221.9	+	+	0	-	-
11	365	+	+	19	-	-	101	-	-	348.6	+	+	193.8	+	+	1.7	-	-
12	369.91	+	+	201.6	+	+	0	-	-	84.9	-	-	300.7	+	+	53.7	-	-
13	452.6	+	+	276.4	+	+	301.5	+	+	126.8	-	-	194.1	+	+	0	-	-
14	383	+	+	0	-	-	199.7	+	+	5.21	-	-	72.94	-	-	32	-	-
15	380	+	+	223	+	+	298.7	+	+	87.9	-	-	10	-	-	0	-	-
16	385	+	+	0	-	-	2	-	-	0	-	-	0	-	-	0	-	-
17	388	+	+	41.26	-	-	0	-	-	198.8	+	+	0	-	-	63	-	-
18	404.2	+	+	150	-	-	200	+	+	135.6	-	-	206	+	+	98.6	-	-
19	410	+	+	315.8	+	+	2	-	-	0	-	-	0	-	-	12	-	-
20	301	+	+	197.8	+	+	33.9	-	-	0	-	-	190	+	+	0	-	-
21	298.5	+	+	14.8	-	-	201	+	+	0	-	-	0	-	-	0	-	-
22	297	+	+	201.8	+	+	12	-	-	194.2	+	+	0	-	-	0	-	-
23	342	+	+	0	-	-	0	-	-	223.7	+	+	0	-	-	19	-	-
24	400	+	+	32	-	-	203.4	+	+	5	-	-	386.8	+	+	0	-	-
25	277	+	+	0	-	-	34	-	-	2	-	-	236.7	+	+	78.2	-	-
26	327.8	+	+	27.8	-	-	197.4	+	+	319	+	+	0	-	-	29.7	-	-
27	359	+	+	0	-	-	76.8	-	-	194.3	+	+	301.4	+	+	5.7	-	-
28	297.6	+	+	278	+	+	0	-	-	11.6	-	-	52.6	-	-	0	-	-
29	341.8	+	+	224.6	+	+	191.5	+	+	97.4	-	-	0	-	-	0	-	-
30	287.9	+	+	0	-	-	221.7	+	+	0	-	-	194.3	+	+	0	-	-
31	197.8	+	+	0	-	-	0	-	-	190	+	+	35.2	-	-	58	-	-
32	398.4	+	+	0	-	-	207.8	+	+	198	+	+	0	-	-	10	-	-
33	320	+	+	12	-	-	0	-	-	0	-	-	207.8	+	+	0	-	-
34	280	+	+	0	-	-	255.4	+	+	0	-	-	0	-	-	0	-	-
35	359	+	+	78	-	-	350.8	+	+	23.7	-	-	194.7	+	+	51.7	-	-
36	337	+	+	202.7	+	+	55	-	-	0	-	-	200.7	+	+	0	-	-
37	408	+	+	400.5	+	+	142.6	-	-	132.1	-	-	97.4	-	-	0	-	-
38	352.6	+	+	194	+	+	0	-	-	200	+	+	2.6	-	-	34.8	-	-
39	295	+	+	128.7	-	-	287.4	+	+	0	-	-	0	-	-	4.5	-	-
40	368	+	+	0	-	-	351.7	+	+	0	-	-	197	+	+	0	-	-
41	401	+	+	289.4	+	+	36.9	-	-	321.7	+	+	0	-	-	0	-	-
42	294	+	+	0	-	-	0	-	-	287	+	+	195.7	+	+	0	-	-
43	281.3	+	+	257.6	+	+	198.9	+	+	79	-	-	0	-	-	36.7	-	-
44	354	+	+	0	-	-	316	+	+	299.8	+	+	19	-	-	0	-	-
45	386	+	+	192.7	+	+	94.6	-	-	0	-	-	380	+	+	0	-	-

**46**	379	+	+	251	+	+	238.4	+	+	24	-	-	1.4	-	-	8.7	-	-
**47**	350	+	+	287.1	+	+	348.7	+	+	0	-	-	15	-	-	98.2	-	-
**48**	355.6	+	+	191.3	+	+	0	-	-	312	+	+	0	-	-	2	-	-
**49**	397.8	+	+	0	-	-	208.1	+	+	0	-	-	0	-	-	51	-	-
**50**	407.6	+	+	148.6	-	-	90	-	-	399.2	+	+	206.4	+	+	101	-	-
**51**	312	+	+	195.3	-	+	56.7	-	-	23	-	-	0	-	-	0	-	-
**52**	349.7	+	+	0	-	-	0	-	-	197.2	+	+	177.8	-	-	56.8	-	-
**53**	401	+	+	138	-	-	12.6	-	-	188	+	-	0	-	-	356	+	+
**54**	396.2	+	+	148.7	+	-	0	-	-	35.8	-	-	0	-	-	0	-	-
**55**	380	+	+	357.9	+	+	151.7	-	-	101.8	-	-	6.8	-	-	0	-	-
**56**	419	+	+	0	-	-	234.3	-	+	13	-	-	2	-	-	136.2	+	-
**57**	202.6	+	+	56	-	-	0	-	-	0	-	-	125.9	-	-	199.6	-	+

S2.3. Statistical analysis of CDP efficacy on Mice models

Table sup4. CDP scores vs. Permanent Cross tabulation for 57 *in-vivo* mice samples

			PERMANENT		
			Negative	Positive	Total
CDP answer	Negative	Count	197	0	197
		% Within CDP answer	100.0%	0.0%	100.0%
		% Within PERMANENT	98.5%	0.0%	57.6%
	Positive	Count	3	142	145
		% Within CDP answer	2.1%	97.9%	100.0%
		% Within PERMANENT	1.5%	100.0%	42.4%
Total		Count	200	142	342
		% Within CDP answer	58.5%	41.5%	100.0%
		% Within PERMANENT	100.0%	100.0%	100.0%

Sensitivity = 100.00%

Specificity = 98.50%

Precision = Positive Predictive Value = 97.9%

Accuracy =Positive Likelihood ratio = 100%

Selectivity=98.50%

Table sup5. FROZEN * PERMANENT Cross tabulation for 57 *in-vivo* mice samples

			PERMANENT		
			Negative	Positive	Total
FROZEN	Negative	Count	197	3	200
		% Within FROZEN	98.5%	1.5%	100.0%
		% Within PERMANENT	98.5%	2.1%	58.5%
	Positive	Count	3	139	142
		% Within FROZEN	2.1%	97.9%	100.0%
		% Within PERMANENT	1.5%	97.9%	41.5%
Total		Count	200	142	342
		% Within FROZEN	58.5%	41.5%	100.0%
		% Within PERMANENT	100.0%	100.0%	100.0%

Sensitivity = 97.9%

Specificity = 98.50%

Precision =Positive Predictive Value = 97.9%

Accuracy =Positive Likelihood ratio = 98.50%

Selectivity=96%

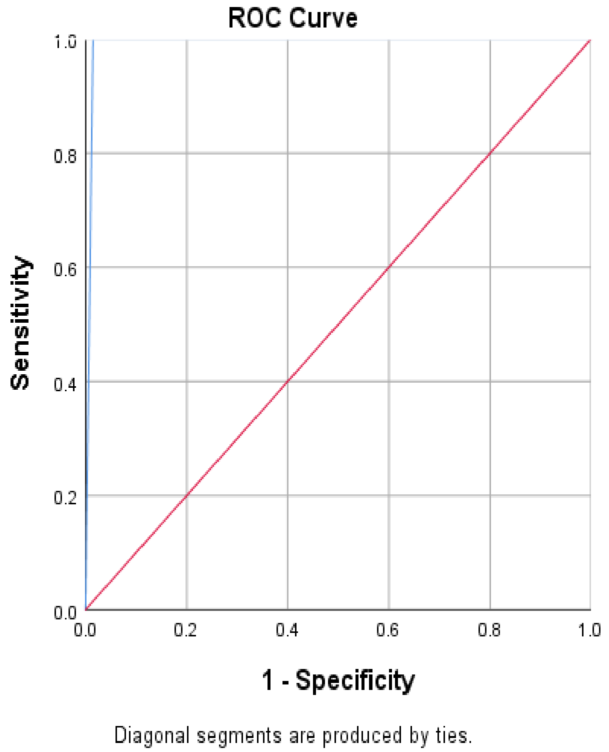

Figure sup7. Receiver Operating Characteristic (ROC) diagram for CDP vs. permanent results in 57 *in-vivo* mice samples

Table sup6. Area Under the Curve (AUC) for CDP vs. permanent results in 57 *in-vivo* mice samples

Test Result Variable(s): CDP answer

Area	Std. Error[a]	Asymptotic Sig.[b]	Asymptotic 99% Confidence Interval	
			Lower Bound	Upper Bound
.992	.005	.000	.980	1.000
(0.992500)	(0.004994)	(2.3799E-54)	(0.979637)	(1.000000)

The test result variable(s): CDP answer has at least one tie between the positive actual state group and the negative actual state group. Statistics may be biased.

a. Under the nonparametric assumption

b. Null hypothesis: true area = 0.5

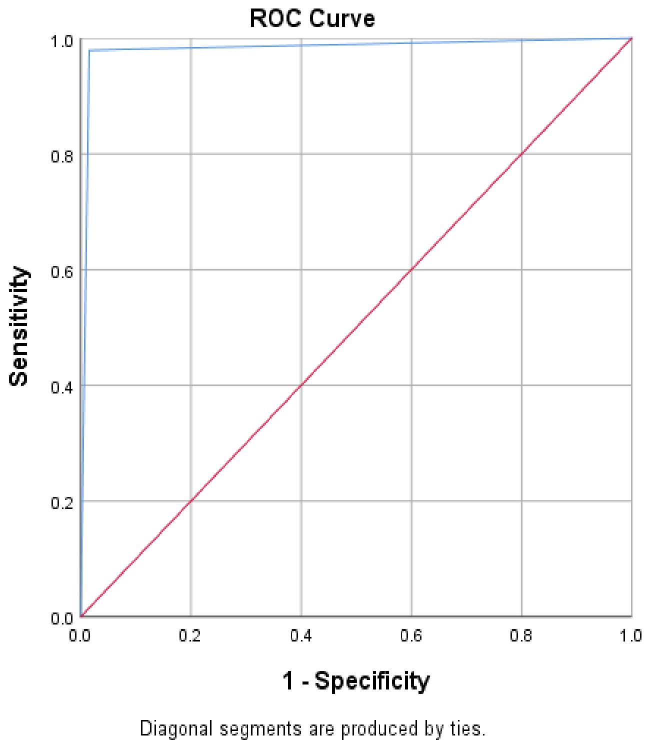

Figure sup8. ROC diagram for frozen vs. permanent results in in 57 *in-vivo* mice samples

Table sup7. AUC table for frozen vs. permanent results in 57 *in-vivo* mice samples

Test Result Variable(s): FROZEN

Area	Std. Error[a]	Asymptotic Sig.[b]	Asymptotic 99% Confidence Interval Lower Bound	Asymptotic 99% Confidence Interval Upper Bound
.982	.009	.000	.960	1.000
(0.981937)	(0.008568)	(4.0432E-52)	(0.959866)	(1.000000)

The test result variable(s): FROZEN has at least one tie between the positive actual state group and the negative actual state group. Statistics may be biased.

a. Under the nonparametric assumption

b. Null hypothesis: true area = 0.5

The permanent pathology is considered the gold standard test for the diagnosis of non-cancerous and cancerous specimens. First, the specificity, sensitivity, positive predictive value, negative predictive value, and likelihood ratios of CDP and frozen have been calculated separately. The

true and false positive and negative data are shown in detail in Supplementary Tables 4&5. It is shown that the accuracy, sensitivity, and selectivity of CDP is better than the frozen pathology.

As a result, to evaluate each of the diagnostic tests, the receiver operating characteristic (ROC) test has been done to compare each of them with the gold standard test (permanent pathology). As it is shown in ROC and Area under the curves (AUC) table for CDP, the area under the curve is 0.992 (P-value<0.0001 and CI99% 0.98-1.00) (Supplementary Fig.7 & Supplementary Table 6), which is higher than 0.9, so the test is appropriate for diagnosis.

The same calculation has been done for frozen, and the result shows that (Supplementary Fig.8 & Supplementary Table 7), the area is 0.982 (P-value<0.0001 and CI99% 0.96-1.00), which shows that the test is a reliable diagnostic test and it has a good balance of sensitivity and specificity.

In conclusion, the CDP has proper sensitivity and accuracy, and it can be used as a diagnostic test of cancerous specimens. Also, the ROC test result shows that the CDP has better results than frozen due to the higher area under the curve of CDP (0.992>0.982). The accurate amount of each index is shown in the tables.

(**Note**. All of the statistical analysis has been done with SPSS v26.)

S3. Pathological classification of CDP responses on *in-vitro* breast samples

In this step, CDP was applied to record the current peaks of 258 *in-vitro* human fresh samples prepared from 74 breast cancer patients. According to an ethically approved protocol (IR.TUMS.VCR.REC.1397.355) at our breast cancer central clinics and assistant hospitals, patients provided consent. Live slices from CNB or surgically removed samples were cut into similar specimens and directly transferred through micro wells contain RPMI-1640 without any preprocessing. Meaningful results were observed after comparing the experimental categorization

of samples through their CDP recorded current peaks with their categorization through their H&E pathological diagnoses (Supplementary Table 8).

Table sup8. Examples of DIN-CDP scored *in-vitro* samples of dissected tissues through surgery or biopsy from 74 breast disease patients proposed based on classification introduced in figure 4.
Pos.: positive sample: ranged from ADH to IDC.
Neg.: Negative sample: ranged from normal breast to low-risk benign lesions,
Neg. (Beware): Negative sample; equal to moderate risk benign lesions
F.Pos.: false positives of CDP score. **F.Neg.:** false negatives of CDP scores.

Sample ID	CDP Score (Peak Current (µA))	H&E (DIN classification diagnosis)	Patient ID	CDP Score (Peak Current(µA))	H&E (DIN classification diagnosis)	Sample ID	CDP Score (Peak Current (µA))	H&E (DIN classification diagnosis)
**1**	Neg. (95)	SA (UDH)	**87**	Pos. (227.4)	ADH (DIN1b)	**173**	Pos. (725.4)	IDC >5%
**2**	Neg. (83.4)	Mild DH (UDH)	**88**	Neg. (87.3)	FCC (UDH)	**174**	Neg. (124)	FCC with typical DH (DIN1a)
**3**	Neg. (112)	FCC with CCC (UDH)	**89**	Pos. (266)	One foci of DCIS (DIN1c)	**175**	Pos. (247.3)	ADH (DIN1b)
**4**	Pos. (340.9)	Low grade DCIS (DIN1c)	**90**	Neg. (9)	Normal breast stroma (Free)	**176**	F.Pos. (201.6)	SA (UDH)
**5**	Pos. (774.3)	Ductal Carcinoma (>DIN3)	**91**	Pos. (389)	Two foci of low-grade DCIS (DIN1c)	**177**	Neg. (114.7)	FCC with CCC (UDH)
**6**	Neg. (153)	Florid DH (DIN1a)	**92**	Neg. (181)	Complex SA (DIN1a)	**178**	Pos. (318.5)	Lobular carcinoma
**7**	Pos. (223.4)	ADH (DIN1b)	**93**	Neg. (100.6)	Non proliferating FCC (UDH)	**179**	Pos. (214.7)	Lobular cancerization (DIN1b)
**8**	Pos. (393)	DCIS (DIN2)	**94**	Neg. (28)	Normal breast stroma (Free)	**180**	Pos. (670)	IDC >5%
**9**	Pos. (371)	Low grade DCIS (DIN1c)	**95**	Neg. (89)	UDH (UDH)	**181**	Pos. (207.6)	ADH (DIN1b)
**10**	Neg. (12.4)	Fatty breast tissue (Free)	**96**	Neg. (110)	SA with FCC (UDH)	**182**	Neg. (87)	Non proliferating FCC (UDH)
**11**	Neg. (87)	Mild DH (UDH)	**97**	F.Pos. (210.3)	Moderate UDH (UDH)	**183**	Neg. (169.3)	Florid DH (DIN1a)
**12**	Pos. (241.7)	Lobular cancerization (DIN1b)	**98**	Neg. (77)	Fatty breast tissue (Free)	**184**	Pos. (203.4)	ADH (DIN1b)
**13**	Pos. (782.6)	Ductal Carcinoma (>DIN3)	**99**	Pos. (741)	Ductal Carcinoma (>DIN3)	**185**	F.Neg. (167.9)	Two foci of ADH (DIN1b)
**14**	Pos. (390)	Low grade DCIS (DIN1c)	**100**	F.Pos. (205)	FCC with CCC (UDH)	**186**	Neg. (127.5)	moderate UDH (DIN1a)
**15**	Pos. (411.7)	Low grade DCIS (DIN1c)	**101**	Neg. (16)	Normal breast stroma (Free)	**187**	Pos. (663)	IDC >5%
**16**	Pos. (820.4)	Ductal Carcinoma (>DIN3)	**102**	Neg. (27)	Fatty breast tissue (Free)	**188**	Pos. (601)	DCIS (DIN3)
**17**	Neg. (92.3)	SA with pseudo infiltration (UDH)	**103**	Pos. (570)	DCIS (DIN3)	**189**	Pos. (211.8)	LIN 2
**18**	Pos. (223)	ADH (DIN1b)	**104**	Neg. (68)	Fatty breast tissue (Free)	**190**	Neg. (116.2)	SA with CCC (UDH)
**19**	Neg. (134.6)	FCC with typical DH (DIN1a)	**105**	Pos. (615)	DCIS (DIN3)	**191**	Neg. (158)	Florid DH (DIN1a)
**20**	Neg. (Beware) (153)	Atypical CCC (DIN1a)	**106**	Neg. (88)	UDH (UDH)	**192**	Pos. (220)	ADH (DIN1b)
**21**	Pos. (630)	DCIS (DIN2)	**107**	Pos. (233.4)	ADH (DIN1b)	**193**	Neg. (129.5)	FCC with typical DH (DIN1a)
**22**	Pos. (814.4)	Ductal Carcinoma (>DIN3)	**108**	Neg. (9)	Fatty breast tissue (Free)	**194**	Pos. (412.8)	DCIS (DIN2)
**23**	Pos. (739)	Ductal Carcinoma (>DIN3)	**109**	Neg. (116)	FCC with CCC (UDH)	**195**	Neg. (8)	Fatty breast tissue (Free)
**24**	Neg. (85.9)	Mild UDH (UDH)	**110**	Neg. (102)	SA (UDH)	**196**	Pos. (277.2)	One foci of DCIS (DIN1c)

25	Neg. (Beware) (173)	Complex fibroadenoma (DIN1a)	111	Neg. (92)	Non proliferating FCC (UDH)	197	Neg. (Be aware) (201.6)	One focus of ADH (DIN1a)
26	Neg. (95.4)	UDH (UDH)	112	Neg. (86.5)	Non proliferating FCC (UDH)	198	Neg. (133.4)	FCC with moderate UDH (DIN1a)
27	Neg. (116)	FCC with CCC (UDH)	113	Neg. (62)	Normal breast stroma (Free)	199	Neg. (116)	FCC with CCC (UDH)
28	Neg. (110.5)	FCC (UDH)	114	F.Neg. (114.2)	Fatty breast tissue (Free)	200	Pos. (721)	IDC >5%
29	Neg. (97.5)	Mild UDH (UDH)	115	Pos. (239.6)	ADH (DIN1b)	201	Pos. (822.7)	Ductal Carcinoma (>DIN3)
30	Pos. (207.8)	ADH (DIN1b)	116	Neg. (Be aware) 192.5	One focus of ADH (DIN1a)	202	Neg. (77.3)	Normal breast stroma (Free)
31	Neg. (113)	FCC with SA (UDH)	117	Pos. (387.4)	DCIS (DIN1c)	203	F.Neg. (113.4)	More than two foci of ADH (DIN1b)
32	Pos. (462)	Phylloids tumor	118	Neg. (120.4)	Moderate UDH (DIN1a)	204	Neg. (53.4)	Normal breast stroma (Free)
33	Pos. (237.6)	Lobular Carcinoma	119	Pos. (241.9)	ADH (DIN1b)	205	Pos. (204.2)	ADH (DIN1b)
34	Pos. (301.5)	Low grade DCIS (DIN1c)	120	Neg. (108.4)	FCC with CCC (UDH)	206	Pos. (769)	IDC >5%
35	Pos. (748)	Ductal Carcinoma (>DIN3)	121	Pos. (744)	IDC >5%	207	Pos. (722.8)	Ductal Carcinoma (>DIN3)
36	Pos. (222)	ADH (DIN1b)	122	F.Pos. (261.8)	Florid DH (DIN1a)	208	Neg. (41.5)	Fatty breast tissue (Free)
37	Neg. (10.4)	Fatty breast tissue (Free)	123	Neg. (Be aware) (142)	Flat epithelial atypia (DIN1a)	209	Pos. (433.5)	DCIS (DIN2)
38	Neg. (Be aware) (167.4)	FCC with florid atypical DH with CCC (DIN1a)	124	Pos. (231.8)	ADH (DIN1b)	210	Pos. (386.1)	More than one foci of DCIS (DIN1c)
39	Neg. (132)	FCC with typical DH (DIN1a)	125	Neg. (102.8)	FCC (UDH)	211	Neg. (118.7)	Moderate UDH (DIN1a)
40	Pos. (799)	Ductal Carcinoma (>DIN3)	126	Neg. (85)	Non proliferating FCC (UDH)	212	Pos. (832.5)	Ductal Carcinoma (>DIN3)
41	Pos. (411.9)	DCIS (DIN1c)	127	Neg. (9.3)	Non proliferating FCC (UDH)	213	Pos. (216)	ADH (DIN1b)
42	Pos. (831.6)	Ductal Carcinoma (>DIN3)	128	F.Pos. (139.7)	SA (UDH)	214	F.Pos. (238.5)	Florid DH (DIN1a)
43	Pos. (822)	Ductal Carcinoma (>DIN3)	129	Pos. (248.5)	ADH (DIN1b)	215	Neg. (84.5)	Non proliferating FCC (UDH)
44	Neg. (88.6)	Mild UDH (UDH)	130	Neg. (88.3)	Non proliferating FCC (UDH)	216	Neg. (2)	Normal breast stroma (Free)
45	Neg. (102)	FCC with Mild UDH (UDH)	131	Neg. (16)	Fatty breast tissue (Free)	217	Neg. (93)	Mild UDH (UDH)
46	Pos. (801.7)	Ductal Carcinoma (>DIN3)	132	Neg. (119.7)	Moderate UDH (DIN1a)	218	Pos. (212.5)	ADH (DIN1b)
47	Neg. (97)	Sclerosing papillary proliferation (UDH)	133	Pos. (630)	IDC >5%	219	Pos. (248)	ADH (DIN1b)
48	Pos. (245)	ADH (DIN1b)	134	Pos. (589.6)	DCIS (DIN3)	220	Neg. (126.8)	FCC with typical DH (DIN1a)
49	Pos. (803.5)	Ductal Carcinoma (>DIN3)	135	Pos. (257.6)	One foci of DCIS (DIN1c)	221	Pos. (641.7)	IDC >5%
50	Pos. (610)	DCIS (DIN3)	136	Neg. (97.9)	FCC with CCC (UDH)	222	F.Neg. (139.2)	More that two foci of ADH (DIN1b)
51	Pos. (343.2)	Lobular Carcinoma	137	Neg. (104)	Non proliferating FCC (UDH)	223	Neg. (56)	Normal breast stroma (Free)
52	Pos. (219.6)	Lobular cancretization	138	F.Pos. (141.2)	FCC with CCC (UDH)	224	Pos. (729.6)	Ductal Carcinoma (>DIN3)
53	Pos. (790.6)	Ductal Carcinoma (>DIN3)	139	Pos. (211.7)	ADH (DIN1b)	225	Pos. (214.3)	LIN 2
54	Neg. (116)	FCC with CCC (UDH)	140	Neg. (Be aware) (184)	One focus of ADH (DIN1a)	226	Neg. (109.5)	FCC with CCC (UDH)

**55**	Neg. (Be aware) (142)	Flat epithelial atypia (DIN1a)	**141**	Neg. (110.7)	FCC with CCC (UDH)	**227**	Pos. (374.3)	More than one focus of DCIS (DIN1c)
**56**	Neg. (34.4)	Fatty breast tissue (Free)	**142**	Pos. (224.3)	ADH (DIN1b)	**228**	Neg. (102)	Non proliferating FCC (UDH)
**57**	Neg. (9)	Fatty breast tissue (Free)	**143**	Neg. (164.8)	Florid DH (DIN1a)	**229**	Pos. (365)	DCIS (DIN2)
**58**	Neg. (110.6)	FCC with mild UDH (UDH)	**144**	Neg. (132.7)	Moderate UDH (DIN1a)	**230**	Neg. (165.4)	Florid DH (DIN1a)
**59**	Pos. (761.4)	Ductal Carcinoma (>DIN3)	**145**	Neg. (115)	FCC with CCC (UDH)	**231**	F.Pos. (229.3)	Moderate UDH (DIN1a)
**60**	Neg. (116)	Mild UDH with CCC (UDH)	**146**	Pos. (763.5)	IDC >5%	**232**	Neg. (83.6)	FCC (UDH)
**61**	Pos. (224)	ADH (DIN1b)	**147**	Pos. (218)	LIN 2	**233**	Neg. (100.7)	Non proliferating FCC (UDH)
**62**	Pos. (396.9)	Phylloids tumor	**148**	Pos. (401.4)	DCIS (DIN1c)	**234**	Neg. (97)	Non proliferating FCC (UDH)
**63**	Pos. (807)	Ductal Carcinoma (>DIN3)	**149**	Neg. (154)	Florid DH (DIN1a)	**235**	Pos. (598.3)	DCIS (DIN3)
**64**	Neg. (84.6)	FCC (UDH)	**150**	Pos. (242)	Papillary lesion with Atypia	**236**	Pos. (800)	IDC >5%
**65**	Neg. (Be aware) (182)	Complex Fibroadenoma (DIN1a)	**151**	Neg. (112.7)	FCC with SA (UDH)	**237**	Neg. (93)	FCC with CCC (UDH)
**66**	Pos. (802.7)	Ductal Carcinoma (>DIN3)	**152**	Neg. (84.7)	Non proliferating FCC (UDH)	**238**	Pos. (479.4)	Low grade DCIS (DIN1c)
**67**	Pos. (830.7)	Ductal Carcinoma (>DIN3)	**153**	Neg. (90.7)	Nnon proliferating FCC (UDH)	**239**	Neg. (129)	Moderate UDH (DIN1a)
**68**	Neg. (115.3)	FCC with CCC (UDH)	**154**	Neg. (116.2)	FCC with CCC (UDH)	**240**	Neg. (Be aware) (138)	Flat epithelial atypia (DIN1a)
**69**	Neg. (94)	FCC with Adenosis (UDH)	**155**	Pos. (364.9)	More than one focus of DCIS (DIN1c)	**241**	Pos. (211)	Lobular cancerization (DIN1b)
**70**	Neg. (5)	Fatty breast tissue (Free)	**156**	Neg. (23.3)	Fatty breast tissue (Free)	**242**	Pos. (284)	Lobular carcinoma
**71**	Pos. (801.6)	Ductal Carcinoma (>DIN3)	**157**	Neg. (89)	Non proliferating FCC (UDH)	**243**	Neg. (103.5)	FCC with CCC (UDH)
**72**	Pos. (519)	DCIS (DIN2)	**158**	Pos. (470.6)	DCIS (DIN3)	**244**	F.Neg. (193.8)	Low grade DCIS(DIN1c)
**73**	Neg. (90)	Mild UDH (UDH)	**159**	F.Neg. (143.5)	Low grade DCIS (DIN1c)	**245**	Neg. (161.5)	Florid DH (DIN1a)
**74**	Neg. (109)	FCC with UDH (UDH)	**160**	Neg. (43)	Normal breast stroma (Free)	**246**	Pos. (219)	LIN 2
**75**	Pos. (244)	ADH (DIN1b)	**161**	Pos. (698)	IDC >5%	**247**	Neg. (99)	Non proliferating FCC (UDH)
**76**	Neg. (185.4)	Low grade DCIS (DIN1c)	**162**	Neg. (113.5)	FCC with CCC (UDH)	**248**	Neg. (96.4)	FCC (UDH)
**77**	Pos. (245)	ADH (DIN1b)	**163**	Pos. (210.5)	ADH (DIN1b)	**249**	Pos. (499.4)	Low grade DCIS (DIN1c)
**78**	Pos. (466.3)	Low grade DCIS (DIN1c)	**164**	Pos. (457.8)	Low grade DCIS (DIN1c)	**250**	Neg. (79)	Normal breast stroma (Free)
**79**	Pos. (213.2)	ADH (DIN1b)	**165**	Neg. (125)	Moderate UDH (DIN1a)	**251**	Neg. (134.2)	FCC with moderate UDH (DIN1a)
**80**	Neg. (92)	FCC (UDH)	**166**	Pos. (260.5)	Papillary lesion with Atypia	**252**	F.Pos. (124.2)	SA (UDH)
**81**	Neg. (37)	Normal breast stroma (Free)	**167**	Pos. (788.4)	IDC >5%	**253**	Neg. (130)	FCC with typical DH (DIN1a)
**82**	F.Pos. (238.1)	One focus of ADH (DIN1a)	**168**	Neg. (33.6)	Fatty breast tissue (Free)	**254**	Neg. (98.6)	FCC with CCC (UDH)
**83**	Pos. (766.3)	Ductal Carcinoma (>DIN3)	**169**	Pos. (236.6)	ADH (DIN1b)	**255**	Neg. (Be aware) (186.8)	One focus of ADH (DIN1a)
**84**	Pos. (377)	Low grade DCIS (DIN1c)	**170**	Neg. (13)	Normal breast stroma (Free)	**256**	Pos. (217.5)	ADH (DIN1b)
**85**	Neg. (84)	SA (UDH)	**171**	Neg. (166.8)	Florid DH (DIN1a)	**257**	Pos. (498)	Low grade DCIS (DIN1c)
**86**	Pos. (747.9)	Ductal Carcinoma (>DIN3)	**172**	Neg. (102)	Non proliferating FCC (UDH)	**258**	Neg. (88.7)	Non proliferating FCC (UDH)

S3.1. Statistical analysis of CDP efficacy on the in-vitro human model

Table sup9. Cross tabulation results for CDP vs. permanent as a gold standard for 258 *in-vitro* samples from 74 patients

			permanent		
			negative	positive	Total
CDPresults	negative	Count	127	11	138
		% within CDPresults	92.0%	8.0%	100.0%
		% within permanent	95.5%	8.8%	53.5%
	positive	Count	6	114	120
		% within CDPresults	5.0%	95.0%	100.0%
		% within permanent	4.5%	91.2%	46.5%
Total		Count	133	125	258
		% within CDPresults	51.6%	48.4%	100.0%
		% within permanent	100.0%	100.0%	100.0%

Sensitivity = 91.00%

Specificity = 95.50%

Precision=Positive Predictive = 95%

Accuracy=Positive Likelihood ratio = 92%

Selectivity=86%

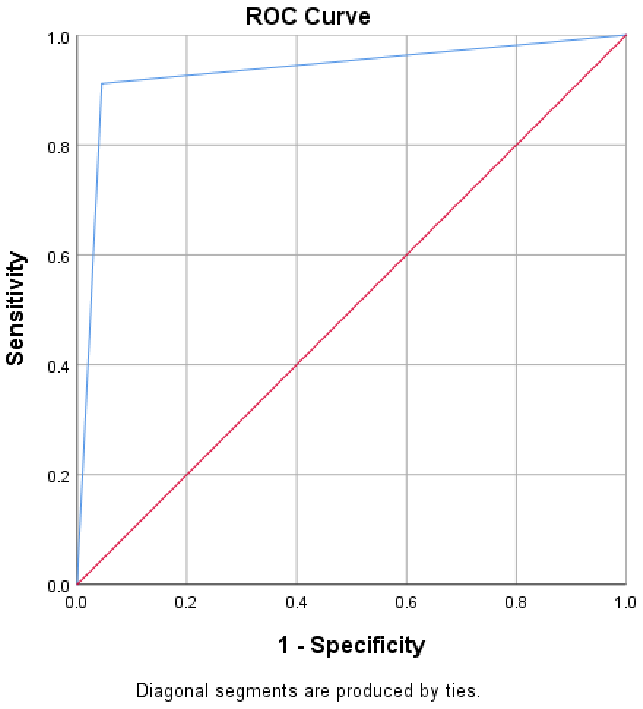

Figure sup9. ROC diagram for CDP vs. permanent results in 258 *in-vitro* samples from 74 patients

Table sup10. AUC for CDP results vs. permanent for 258 *in-vitro* samples from 74 patients

Test Result Variable(s): CDPresults

Area	Std. Error[a]	Asymptotic Sig.[b]	Asymptotic 99% Confidence Interval	
			Lower Bound	Upper Bound
.933	.018	.000	.887	.980

The test result variable(s): CDPresults has at least one tie between the positive actual state group and the negative actual state group. Statistics may be biased.

a. Under the nonparametric assumption

b. Null hypothesis: true area = 0.5

The permanent pathology had been considered as the gold standard test for diagnosis the cancerous specimens. First, the specificity, sensitivity, positive predictive value, negative predictive value, and likelihood ratios of CDP have been calculated. The true and false positive and negative data are shown in detail in Supplementary Table 9.

As a result, to evaluate CDP as a diagnostic tool, the ROC test has been done to compare CDP results with the gold standard test (permanent pathology). As it is shown in the ROC curve (Supplementary Fig.9) and AUC table for CDP (Supplementary Table 10), the area is 0.933 (P-value<0.0001 and CI99% 0.887-0.980), which shows that the test is a reliable diagnostic test, and it has a good balance of sensitivity and specificity.

In conclusion, the CDP has proper sensitivity and specificity and it can be used as a diagnostic test of cancerous specimens.

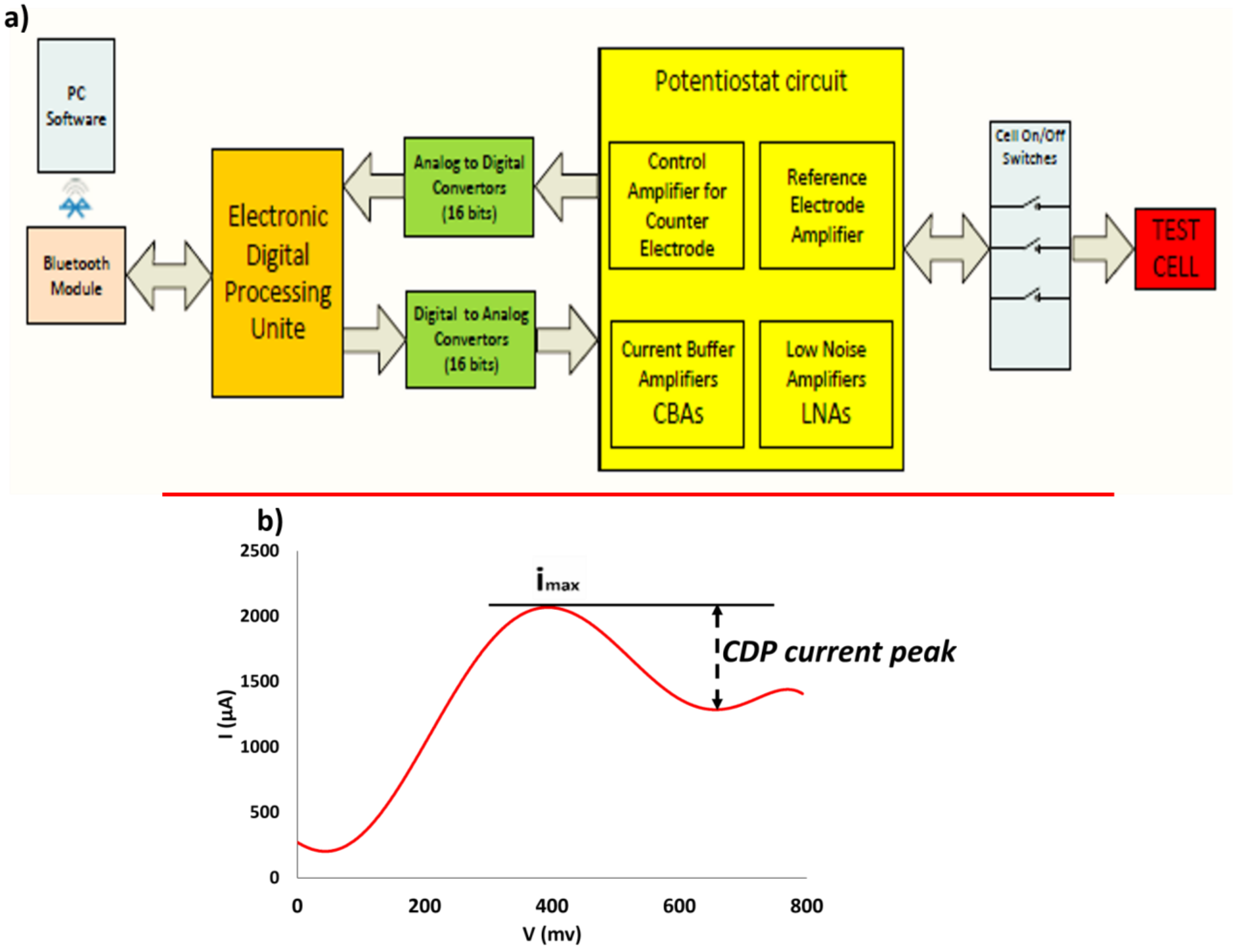

Figure Sup10. The Schematic of an integrated portable automatic electrochemical readout board. Bottom panel: Schematic of the calculating procedure of CDP response value.

Table sup11. Quick-Reference BRISQ Summary/Checklist.

**Bio specimen type**	Solid tissue, Core needle biopsy and surgery from breast tumor
**Anatomical site**	Breast
**Disease status of patients**	Breast Cancer
**Clinical characteristics of patients**	Patients suspicious to breast cancer
**Vital State of patients**	A live
**Clinical diagnosis of patients**	Breast cancer

**Pathology diagnosis**	Ductal Invasive Carcinoma with different grades<br>Lactational changes<br>Adenosis benign glandular proliferation<br>Hyperplasia and inflammation<br>Breast cancer with lymph vascular invasion
**Collection mechanism**	Core needle biopsy and surgery
**Type of stabilization**	For CNT electrochemical sensor: RPMI-1640, 37 °C<br>For Pathological assaying: Fixing in formalin
**Type of long-term preservation**	Freezing for pathological assay, exposing to RNase & storing in liquid N2 for Real Time PCR
**Constitution of preservative**	10% neutral-buffered formalin
**Storage temperature**	37 °C for CNT electrochemical assays
**Storage duration**	After resecting the tumor, it must be maintained in RPMI-1640 medium and held in incubator for 24hr.
**Shipping temperature**	37 °C for CNT electrochemical assay, -196 °C for Real Time PCR
**Composition assessment & selection**	Patients candidate for core needle biopsy were suspicious to cancer candidates for surgery were post biopsied patients.

Table sup12. Oligonucleotide primer and probe sequences used in the present study.

	Sequence (5'->3')	Length	Tm	GC%	Product size (mRNA)	GC% of product
**HIF1A-F**	CGTGTTATCTGTCGCTTTGAGTC	23	59.94	47.83	103	40
**HIF1A-R**	TGTTCCATTTTTCGCTTTCTCTGAG	25	60.28	40.00		
**MYC-F**	CCTCGGATTCTCTGCTCTCC	20	59.33	60.00	161	59.6
**MYC-R**	AGCCTGCCTCTTTTCCACAG	20	60.25	55.00		
**HK2-F**	CAGAAGGTGGAGATGGAGAATCAG	24	60.44	50.00	95	53.7
**HK2-R**	AGGCATTCGGCAATGTGGTC	20	61.32	55.00		
**PGAM1-F**	GGGGAAACGTGTACTGATTGC	21	59.53	52.38	99	55.6
**PGAM1-R**	AGGTTCAGCTCCATGATAGCC	21	59.58	52.38		

**LDHA-F**	ATTCAGCCCGATTCCGTTACC	21	60.47	52.38	129	51.9
**LDHA-R**	CACCAGCAACATTCATTCCACTC	23	60.37	47.83		
**PDK1-F**	TGAAAATGCTAGGCGTCTGTG	21	58.92	47.62	101	38.6
**PDK1-R**	CCACTTGTATTGGCTGTCCTG	21	58.91	52.38		

Reference

[1] J. Zhang *et al.*, "Nondestructive tissue analysis for ex vivo and in vivo cancer diagnosis using a handheld mass spectrometry system," *Sci. Transl. Med.*, vol. 9, no. 406, p. eaan3968, 2017.

[2] M. Jermyn *et al.*, "Intraoperative brain cancer detection with Raman spectroscopy in humans," *Sci. Transl. Med.*, vol. 7, no. 274, pp. 274ra19-274ra19, 2015.

[3] E. Belykh *et al.*, "Utilization of intraoperative confocal laser endomicroscopy in brain tumor surgery.," *J. Neurosurg. Sci.*, vol. 62, no. 6, pp. 704–717, 2018.

[4] M. Thill, "MarginProbe®: intraoperative margin assessment during breast conserving surgery by using radiofrequency spectroscopy," *Expert Rev. Med. Devices*, vol. 10, no. 3, pp. 301–315, 2013.

[5] D. E. Palmer-Toy, D. A. Sarracino, D. Sgroi, R. LeVangie, and P. E. Leopold, "Direct acquisition of matrix-assisted laser desorption/ionization time-of-flight mass spectra from laser capture microdissected tissues," *Clin. Chem.*, vol. 46, no. 9, pp. 1513–1516, 2000.

[6] J. M. Goran, E. N. H. Phan, C. A. Favela, and K. J. Stevenson, "H_2O_2 detection at carbon nanotubes and nitrogen-doped carbon nanotubes: oxidation, reduction, or disproportionation?," *Anal. Chem.*, vol. 87, no. 12, pp. 5989–5996, 2015.

[7] Y. Zhou, Y. Fang, and R. P. Ramasamy, "Non-covalent functionalization of carbon nanotubes for electrochemical biosensor development," *Sensors*, vol. 19, no. 2, p. 392, 2019.

[8] Q. K. Li and W. E. Khalbuss, "Gastrointestinal and Bile Duct Brushing Cytology," in *Diagnostic Cytopathology Board Review and Self-Assessment*, Springer, 2015, pp. 521–571.
